# ATXN2 is a target of N-terminal proteolysis

**DOI:** 10.1371/journal.pone.0296085

**Published:** 2023-12-21

**Authors:** Monika Chitre, Patrick Emery

**Affiliations:** 1 Department of Neurobiology, University of Massachusetts Chan Medical School, Worcester, Massachusetts, United States of America; 2 Interdisciplinary Graduate Program, Morningside Graduate School of Biomedical Sciences, University of Massachusetts Chan Medical School, Worcester, Massachusetts, United States of America; University of Chicago, UNITED STATES

## Abstract

Spinocerebellar ataxia 2 (SCA2) is a neurodegenerative disorder caused by the expansion of the poly-glutamine (polyQ) tract of Ataxin-2 (ATXN2). Other polyQ-containing proteins such as ATXN7 and huntingtin are associated with the development of neurodegenerative diseases when their N-terminal polyQ domains are expanded. Furthermore, they undergo proteolytic processing events that produce N-terminal fragments that include the polyQ stretch, which are implicated in pathogenesis. Interestingly, N-terminal ATXN2 fragments were reported in a brain extract from a SCA2 patient, but it is currently unknown whether an expanded polyQ domain contributes to ATXN2 proteolytic susceptibility. Here, we used transient expression in HEK293 cells to determine whether ATXN2 is a target for specific N-terminal proteolysis. We found that ATXN2 proteins with either normal or expanded polyQ stretches undergo proteolytic cleavage releasing an N-terminal polyQ-containing fragment. We identified a short amino acid sequence downstream of the polyQ domain that is necessary for N-terminal cleavage of full-length ATXN2 and sufficient to induce proteolysis of a heterologous protein. However, this sequence is not required for cleavage of a short ATXN2 isoform produced from an alternative start codon located just upstream of the CAG repeats encoding the polyQ domain. Our study extends our understanding of ATXN2 posttranslational regulation by revealing that this protein can be the target of specific proteolytic cleavage events releasing polyQ-containing products that are modulated by the N-terminal domain of ATXN2. N-terminal ATXN2 proteolysis of expanded polyQ domains might contribute to SCA2 pathology, as observed in other neurodegenerative disorders caused by polyQ domain expansion.

## Introduction

Spinal cerebellar ataxia (SCA2) is an autosomal-dominant, progressive, late-onset neurodegenerative disease that predominantly affects neurons in the cerebellum, brain stem, and spinal cord [1]. Patients typically present with progressive cerebellar ataxia, ocular symptoms such as nystagmus or slow saccadic eye movements, and other neurological deficits such as peripheral neuropathy. SCA2 is associated with the expansion of the CAG-repeat region of the *ataxin 2* (*ATXN2)* gene and the resultant lengthened poly-glutamine (polyQ) tract that is located near the N-terminus of the ATXN2 protein [1–4]. In the general population, the polyQ stretch usually contains 22 glutamines. Repeat lengths greater than 34 glutamines typically cause SCA2 [1–4]. In addition, intermediate expansions of 27–33 glutamine repeats are associated with the development of amyotrophic lateral sclerosis (ALS) [5] and in some populations, Parkinson’s disease (PD) [6, 7].

The best characterized biochemical function of the ATXN2 protein is the regulation of mRNA stability and translation, including via miRNA silencing [8–14]. ATXN2 is present in and regulates the formation of stress granules and P-bodies, which also control mRNA metabolism and translation [15–17]. Many mRNA regulatory pathways have been implicated in the development of neurodegenerative diseases, with changes in polyQ length of specific proteins being a common feature [18]. Indeed, polyQ lengthening is thought to influence protein-protein interactions, thus altering protein function and resulting in the formation of protein aggregates that might themselves be toxic.

To understand the role of ATXN2 in the development of neurodegenerative diseases, it is important to study its function as well as its regulation at both the gene transcription, mRNA and protein levels. Indeed, these mechanisms may prove to be promising therapeutic targets for reducing toxic protein loads. Transcriptionally, *ATXN2* expression is regulated by the transcription factor ETS1 [19]. Interestingly, *ATXN2* pre-mRNA transcripts can produce multiple splice variants, but whether alternatively spliced *ATXN2* transcripts encode ATXN2 isoforms with differential activity or specific function is unclear [20]. Furthermore, two potential start codons in the same translational frames are present in the 5’ end of the *ATXN2* gene and might thus add to isoform complexity [19]. Post-translational regulation of ATXN2 has also been studied. For example, phosphorylation of ATXN2 by the cyclin-dependent kinase 5-p25 (Cdk5–p25) targets ATXN2 protein for degradation [21].

PolyQ proteins implicated in neurodegeneration can undergo an additional form of post-translational processing that could be targeted for therapeutic benefit. In the case of Huntingtin (Htt) [22] and ATXN7 [23], N-terminal proteolysis occurs near the N-terminal polyQ domain, thus releasing a polyQ containing fragment. Additionally, ATXN3 undergoes both N- and C-terminal proteolysis that produces N-terminal fragments as well as C-terminal fragments that contain its C-terminal polyQ domain [24, 25]. Various proteases such as caspases, calpains, or matrix metalloproteinases target these polyQ proteins for cleavage [18, 23, 26]. Strikingly, Huntington’s disease can be modeled in transgenic mice expressing the N-terminal fragment of mutant human Htt, suggesting that N-terminal cleavage products can be toxic [27]. Moreover, inhibition of the protease that targets ATXN3 [25] or expression of cleavage-resistant forms of Htt [28] or ATXN7 [23] delays disease progression in animal models.

Despite the presence of an N-terminal polyQ domain in ATXN2, it has yet to be determined whether ATXN2 might also be the target of specific N-terminal proteolysis. Interestingly, an early study using brain extract from a SCA2 patient detected polyQ containing ATXN2 fragments, implicating N-terminal proteolysis of ATXN2 [29]. Here, we present evidence that ATXN2 indeed can undergo specific N-terminal proteolytic cleavage, irrespective of polyQ length. This was the case for both long and short ATXN2 isoforms produced from alternative ATG start codons. We precisely mapped the cleavage site for the long isoform to a short and highly conserved motif located just C-terminal of the polyQ domain.

## Material and methods

### Plasmid generation, preparation and sequence validation

ATG1-*ATXN2*-22Q and ATG1-*ATXN2*-39Q inserted in the pcDNA6 vector were generously gifted by Dr. Aaron Gitler (Stanford University) [5], and served as starting points for our cloning strategies. To generate novel pcDNA6-based expression constructs, we used either classic cloning approaches with various restriction enzymes and T4 DNA ligase, or generated fragments with overlapping sequences, which were then combined with the NEBuilder Hifi DNA Assembly Kit (New England Biolabs #E5520S). In most cases, we used standard PCR protocols with Taq, Phusion, or Q5 polymerases (New England Biolabs #M0273S,#M0530S, or #M0491S) to generate various vector and insert fragments. However, for CAG-repeat containing fragments, we used a Taq polymerase-based PCR protocol modified from Smith *et al*. [30] that is specifically optimized to amplify GC-rich regions. These PCR reactions contained 10–20 ng of plasmid, 5% DMSO, 1M betaine, 0.10 μM primer mix, and 2.5 mM MgCl_2_. PCR-cycles consisted of 10 minutes of denaturation at 95°C, 9 minutes of annealing at 62°C and a 10-minute extension step.

**Fig 1:**
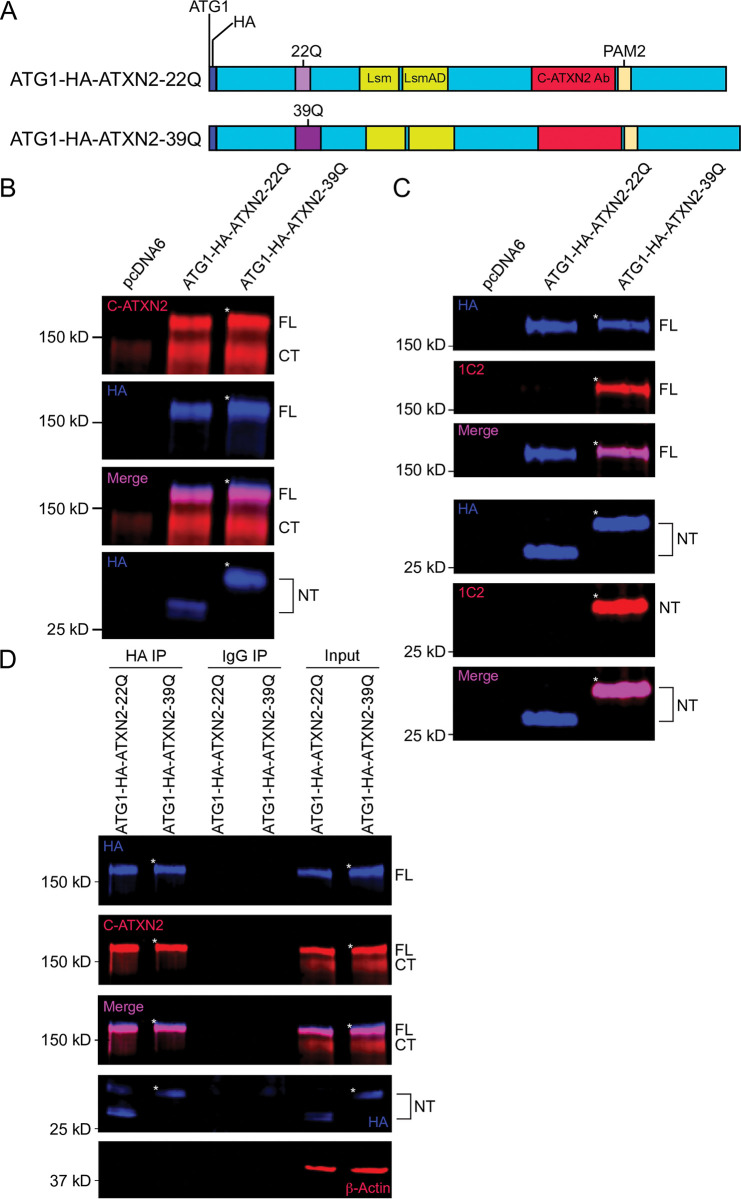
ATXN2 is a target of N-terminal proteolysis when transiently overexpressed in HEK293E cells. **A.** Schematic of HA-tagged full-length (1313 amino acid long) wild-type (22Q) and mutant (39Q) ATXN2 proteins, produced in HEK293 cells from a pcDNA6 vector with a CMV promoter. An N- terminal tag (dark blue) was inserted immediately after the 1st ATG (ATG1) for both the WT (ATG1-HA-ATXN2-22Q, polyQ in light purple) and mutant (ATG1-HA-ATXN2-39Q, polyQ in dark purple) forms of ATXN2. **B.** HEK293E cells were transfected with empty vector (pcDNA6), WT (ATG1-HA-ATXN2-22Q) or mutant (ATG1-HA-ATXN2- 39Q) ATXN2 constructs, and cell lysates were analyzed by immunoblotting. Blue: Anti-HA; Red: Anti-C-terminal ATXN2; Magenta: co-localized bands. A Western blot representative of four independent experiments is shown. **C.** HEK293E cells were transfected with either empty vector, ATG1-HA-ATXN2-22Q or ATG1-HA-ATXN2-39Q encoding vectors, and cell lysates were analyzed by immunoblotting. Blue: Anti-HA; Red: Anti-1C2; Magenta: co-localized bands. A Western blot representative of three independent experiments is shown. **D.** Proteins were immunoprecipitated after 48 hours of transfection from cell lysates as described in Methods section. Lysates were analyzed with immunoblotting after immunoprecipitation. Blue: Anti-HA; Red: Anti-C-ATXN2; Magenta: co-localized bands. A Western blot representative of three independent experiments is shown. Image brightness and contrast were adjusted for better visualization of the small fragment in panel D. * signs indicate the slower migration of mutant full-length and N-terminal ATXN2. FL = full-length ATG1-HA-ATXN2 protein; CT = C-terminal fragment; NT = N-terminal fragment.

**Fig 2:**
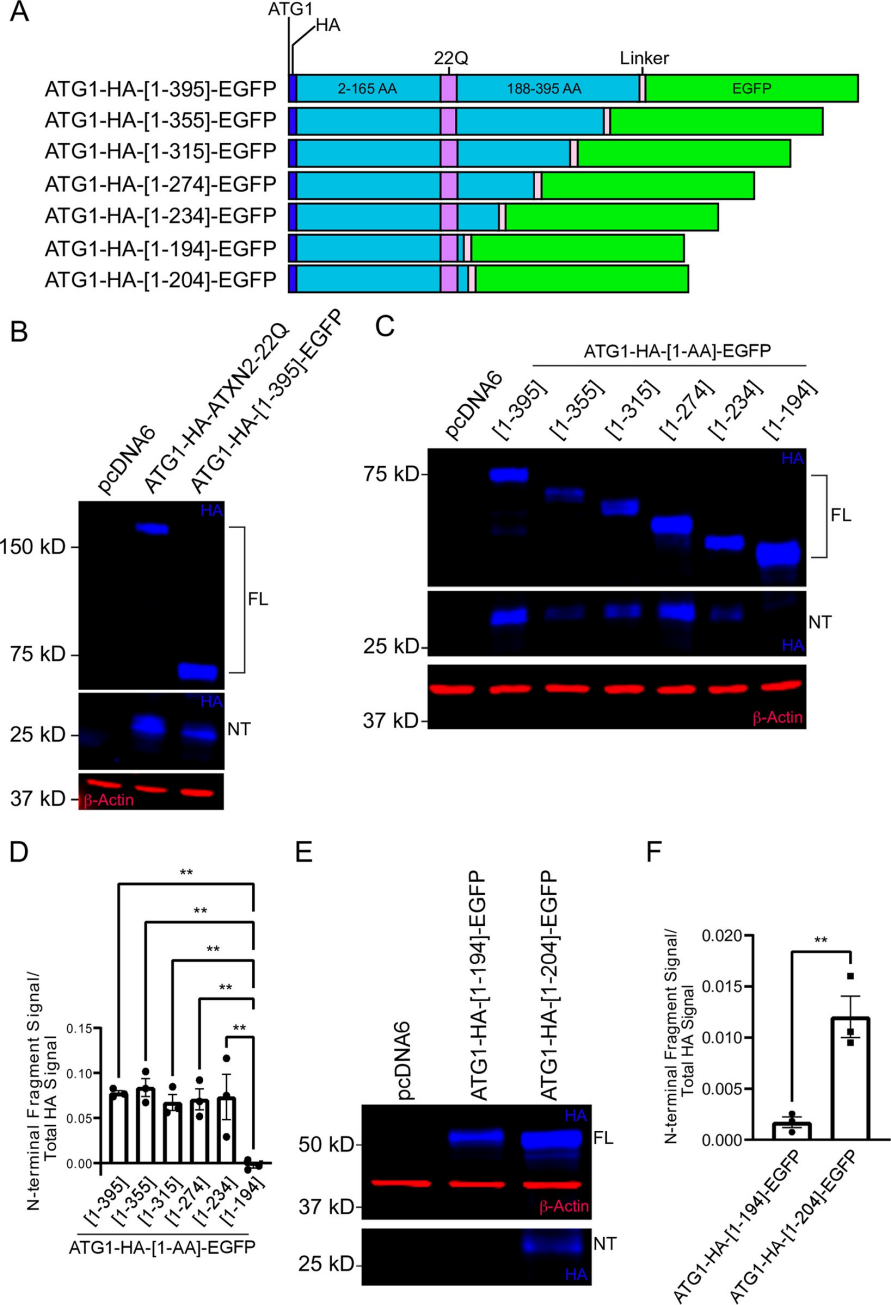
N-terminal cleavage of ATXN2 is abolished after deletion of amino acids downstream of the polyQ domain. **A.** Schematics of proteins expressed in HEK293 cells that comprise ATXN2 N-terminal fragments of various lengths fused to a flexible amino acid linker sequence (light pink) and a C-terminal EGFP reporter (green). **B.** HEK293E cells were transfected with either empty vector, ATG1-HA-ATXN2-22Q or ATG1-HA-[1–395]-EGFP, and cell lysates were analyzed by immunoblotting. Blue: Anti-HA; Red: Anti- β-Actin. FL = full-length ATG1-HA-ATXN2 protein and full-length ATG1-HA-[1–395]-EGFP fusion protein; NT = N-terminal fragment. A Western blot representative of three independent experiments is shown. **C.** HEK293E cells were transfected with different ATG1-HA-[1-AA]-EGFP constructs, with AA symbolizing the variable position of the most C-terminal ATXN2 amino acid included in the constructs. Cell lysates were analyzed by immunoblotting. Blue: Anti-HA; Red: Anti- β-Actin. FL = Full-length ATG1-HA-[1-AA]-EGFP fusion proteins; NT = N-terminal fragment. A Western blot representative of three independent experiments is shown. **D.** Quantification of N-terminal cleavage fragment ratio (signal intensity of N-terminal fragment divided by total HA signal). Statistical analysis performed with one-way ANOVA with Dunnett’s multiple comparisons test. n = 3, **P < 0.01 **E.** HEK293E cells were transfected with ATG1-HA-[1–194]-EGFP or ATG1-HA-[1–204]-EGFP, and cell lysates were probed by immunoblotting. Blue: Anti-HA; Red: Anti- β-Actin. FL = Full-length ATG1-HA-[1–194]-EGFP or ATG1-HA-[1–204]-EGFP fusion proteins; NT = N-terminal fragment. A Western blot representative of three independent experiments is shown. **F.** Quantification of N-terminal cleavage fragment ratio. Statistical analysis performed with unpaired, two-tailed T-test. n = 3, **P < 0.01 Image brightness and contrast were adjusted for better visualization of the small fragment in both panel C and E, as cleavage efficiency was quite low in these experiments.

**Fig 3:**
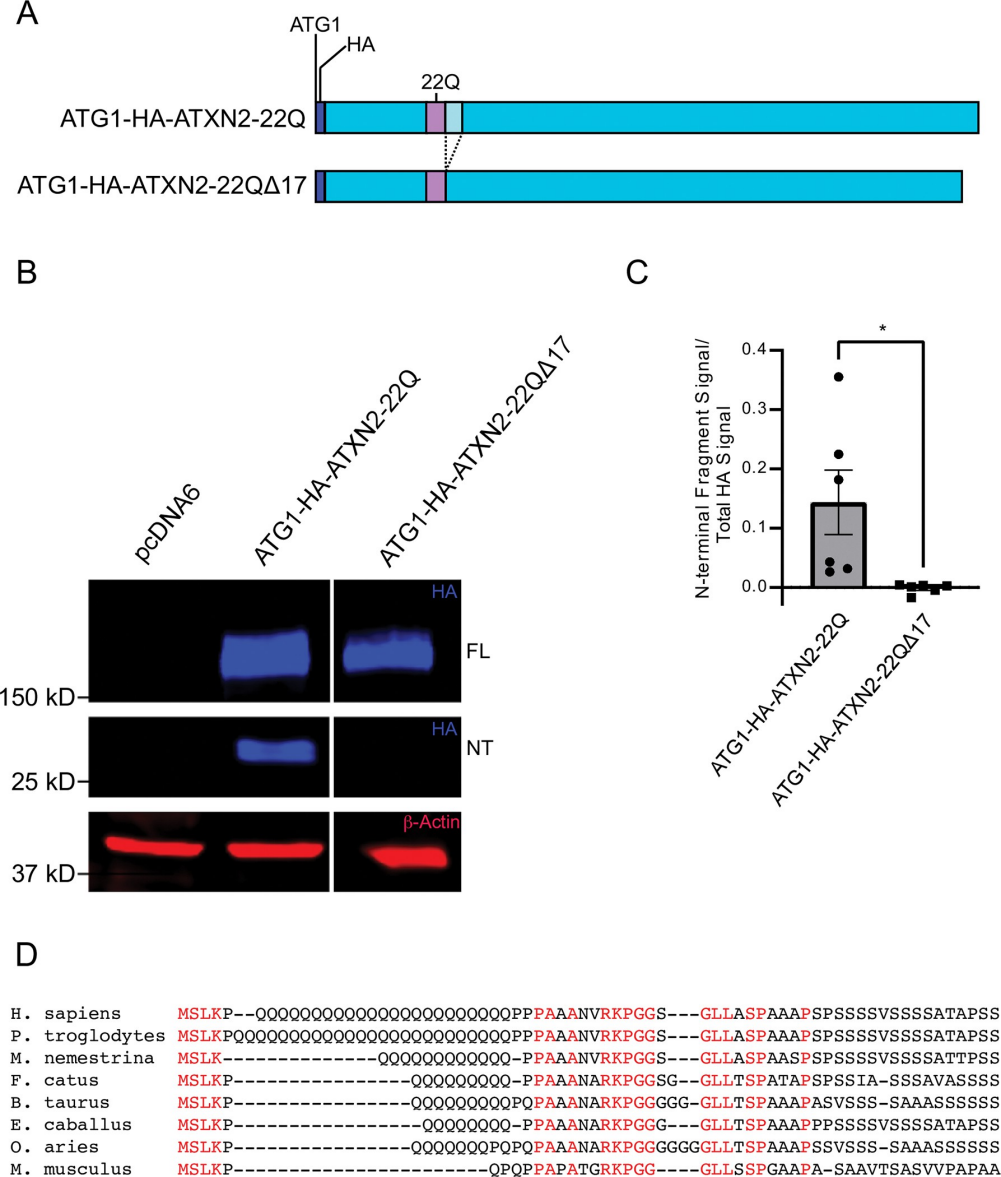
The 17 amino acids downstream of the ATXN2 polyQ are necessary for N-terminal cleavage. **A.** Schematic of the 17 amino acid deletion downstream of the polyQ domain (aqua box). **B.** HEK293E cells were transfected with ATG1- HA-ATXN2-22Q or ATG1-HA-ATXN2-22Q Δ17, and cell lysates analyzed by immunoblotting. Blue: Anti-HA; Red: Anti- β-Actin. FL = Full-length ATG1-HA-ATXN2-22Q or ATG1-HA-ATXN2-22Q Δ17 proteins; NT = N-terminal fragment. A Western blot representative of six independent experiments is shown. **C.** Quantification of N-terminal cleavage fragment ratio. Statistical analysis performed with unpaired, two-tailed T-test. n = 6, P < 0.05. **D.** Sequence alignment showing conservation of the amino acids immediately following the polyQ domain in several mammalian species.

**Fig 4:**
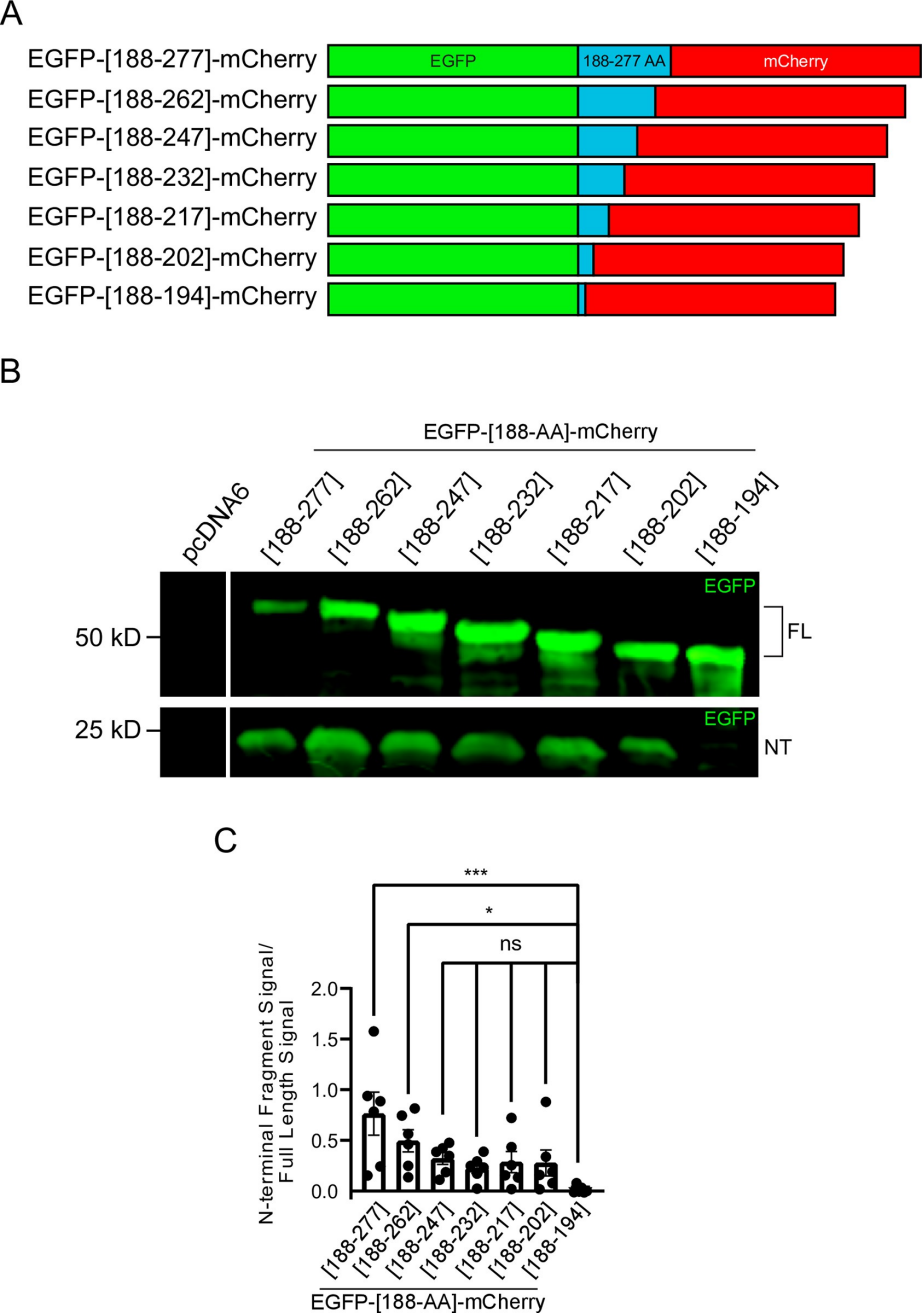
The 17 amino acids downstream of the ATXN2 polyQ are sufficient for proteolytic cleavage to occur. **A.** Schematic of fusion proteins between N- terminal EGFP and C-terminal mCherry moieties with ATXN2 amino acid sequence acting as a linker. 15 amino acids are serially deleted from the ATXN2 sequence in the C to N direction starting from 277aa up to 194aa. **B.** HEK293E cells were transfected with EGFP-[188-AA]-mCherry, and cell lysates were analyzed by immunoblotting. Green = Anti-EGFP. FL = Full-length EGFP-[188-AA]-mCherry fusion proteins; NT = N-terminal fragment. Western blot representative of six independent experiments is shown. **C**. Quantification of cleavage fragment ratio and statistics with EGFP-[188–194]-mCherry being compared to the other constructs. Statistical analysis performed with one-way ANOVA with Dunnett’s multiple comparisons test. n = 6, *P < 0.05, **P < 0.01, and ****P < 0.0001.

**Fig 5:**
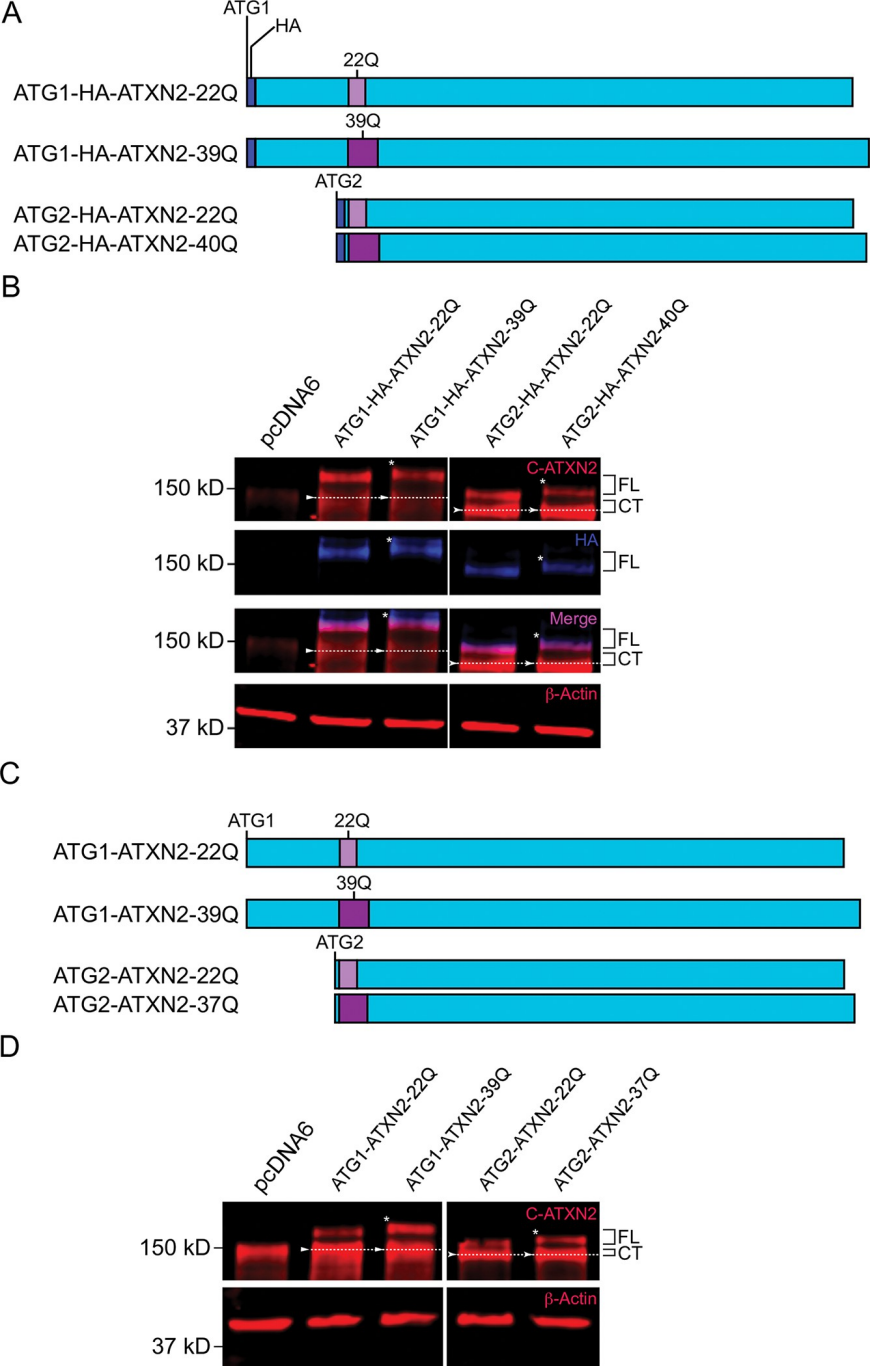
The two HA-ATXN2 isoforms resulting from alternative initiation codon usage undergo N-terminal proteolysis in HEK293E cells. **A.** Schematics of HA-tagged full-length ATXN2 starting at the 1st methionine codon (ATG1) or at the 2nd known start codon (ATG2) that were expressed in HEK293E cells. **B.** HEK293E cells were transfected with plasmids shown in panel A, or with empty plasmid (pcDNA6), and cell lysates were analyzed by immunoblotting. Blue: Anti-HA; Red: Anti-C-ATXN2 or β-Actin; Magenta: co-localized bands. A Western blot representative of three independent experiments is shown. **C.** Schematics for the untagged ATG1 versus the ATG2 ATXN2 translation isoforms. **D.** HEK293E cells were transfected with plasmids shown in panel C, or with empty plasmid (pcDNA6), and cell lysates were analyzed by immunoblotting. Red: Anti-C-ATXN2 or β-Actin. A Western blot representative of three independent experiments is shown. Image brightness and contrast were adjusted in both panel B and D for better visualization of full-length (ATG1) ATXN2 proteins, which were expressed at lower levels than the shorter (ATG2) proteins. FL = Full-length ATG1(-HA)-ATXN2 or ATG2(-HA)-ATXN2 proteins; CT = C-terminal fragment. * signs indicate the slower migration of mutant full-length ATXN2 proteins; Arrowheads and dotted lines, positioned in the middle of bands, emphasize migration difference between ATG1-HA-ATXN2 and ATG2-HA-ATXN2 C-terminal fragments. Flat arrowheads: ATG1-HA-ATXN2 C-terminal fragment; Curved arrowheads: ATG2-HA-ATXN2 C-terminal fragment.

**Fig 6:**
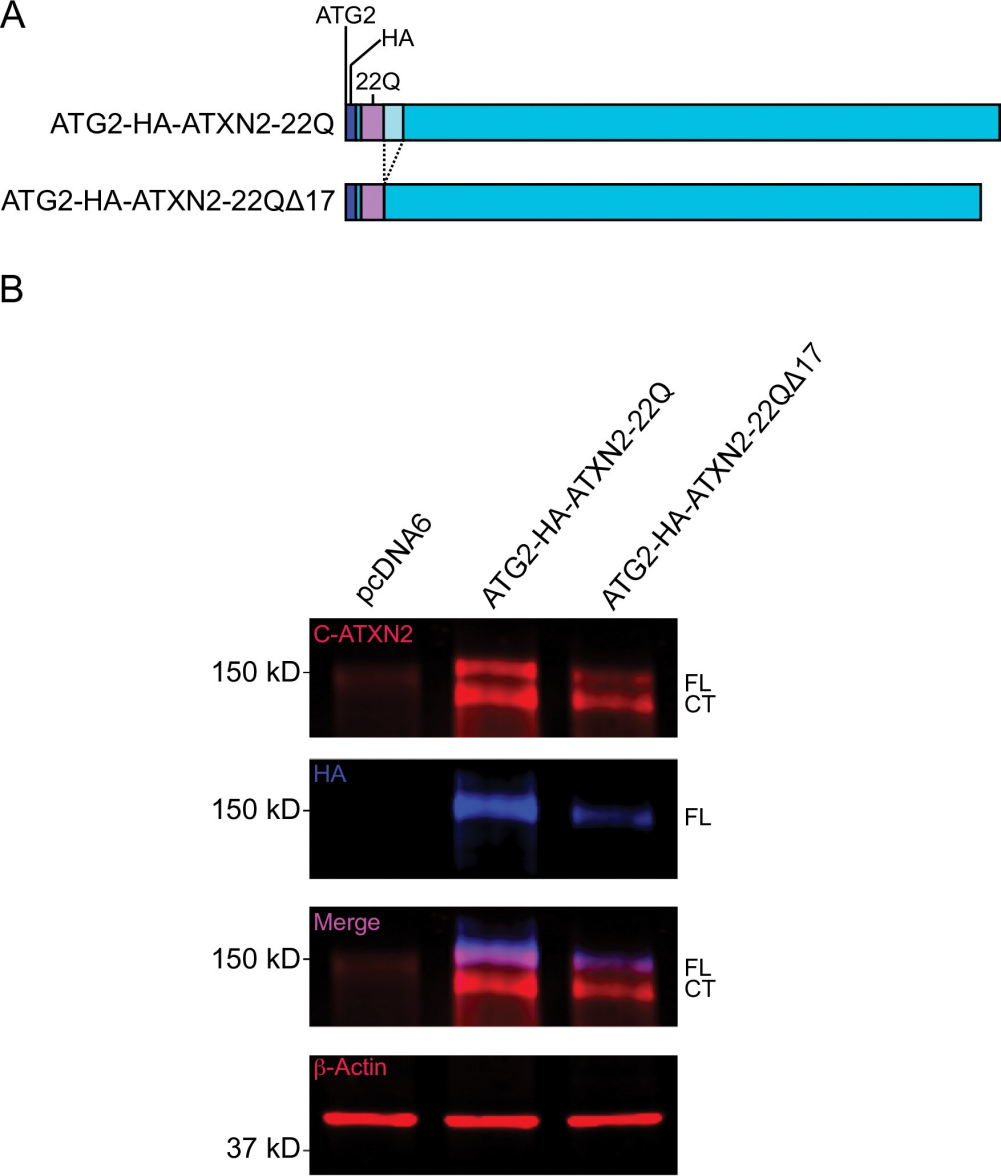
The 17 amino acid sequence downstream of the polyQ domain is not required for cleavage of the shorter ATXN2 translation isoform. **A.** Schematic of the 17 amino acid deletion downstream of polyQ domain from ATG2-HA- ATXN2-22Q sequence. **B.** HEK293E cells were transfected with ATG2-HA-ATXN2-22Q or ATG2-HA-ATXN2-22Q Δ17, and cell lysates were analyzed by immunoblotting. Blue: Anti-HA; Red: Anti-C-ATXN2 or β-Actin; Magenta: co-localized bands. FL = Full-length ATG2-HA-ATXN2-22Q or ATG2-HA-ATXN2-22Q Δ17 proteins; CT = C-terminal fragment. A Western blot representative of four independent experiments is shown.

The coding regions amplified by PCR and inserted in the plasmids were fully sequenced to check for sequence accuracy. For two constructs, we only recovered clones that had slightly altered CAG repeats. Construct ATG2-*ATXN2*-37Q was missing two CAG repeats. We used this construct since a 37Q stretch is pathological [3, 4, 31]. ATG2-*HA*-*ATXN2*-40Q carried an extra CAG. Plasmids were prepared for transfection using the QIAprep Spin Miniprep Kit (Qiagen #27104). Schematics of the proteins encoded by all the pcDNA6 expression constructs used in this paper are shown in the figures. The coding sequences for the HA-tagged ATG1 and ATG2 ATXN2 proteins (Figs 1, 3, 5 and 6) are localized between the Mlu and XbaI restriction sites of pcDNA6, for the N-terminus-EGFP fusions (Fig 2) between KpnI and XbaI, and for the EGFP-mCherry fusions (Fig 4) between KpnI and XbaI.

### Cell culture and transfection

Cells were maintained at 37°C, 5% CO_2_. HEK293E and HEK293T cells were grown in Dulbecco’s modified Eagle’s medium (Gibco #11995065) supplemented with 10% heat-inactivated fetal bovine serum, 2 mM GlutaMAX (Gibco #35050061), and 100 units/mL penicillin-streptomycin.

HEK293E cells were plated in 12-well plates with ~250,000 cells/well. For each transfection except for the various N-terminus-EGFP fusions, 150ng of plasmid was combined with 1.5μl of Mirus-LT1 Transfection Reagent (Mirus Bio #MIR 2300) and 125μl of OptiMEM Reduced Serum Medium (Gibco #31985–070). For the N-terminus-EGFP fusions, the following amounts of plasmid DNA were transfected to adjust protein levels: 150ng of pcDNA6, 300ng of [1–395], 300ng of [1–355], 150ng of [1–315], 50ng of [1–234], and 150ng of [1–194]. After 15 minutes of incubation, each individual transfection mix was added to a single well. Cells were collected and lysed 48–72 hours later.

### Lentiviral transduction

#### ATXN2 shRNA viral media

Viral supernatants with shRNAs targeting human *ATXN2* were obtained from the UMass Chan shRNA Library Core Facility. Each viral supernatant featured an *ATXN2* shRNA in a lentiviral GIPZ vector from Horizon Discovery (Cambridge, UK). shRNA clone IDs and mature antisense sequences were as follows: V2LHS_31547, AGAAAGAAGGGCTTGTCTC; V2LHS_31552, TTCTAGGCCACTGGATATG; V3LHS_392619, TTCTTTAAATCATCAATCT; V3LHS_392620, TTGTTTAGTAGTTGATCCA; V3LHS_392623, TCAATTTTGTCTTTGATCA. In addition to the *ATXN2* shRNA viral supernatants, empty GIPZ vector, non-silencing, and *GAPDH* shRNA were obtained as controls.

#### Lentiviral transduction

1 x 10^6^ HEK293E cells per well were plated in a 6-well dish. The following day, 3mL of viral media was added for each viral supernatant to separate individual wells in a 6-well dish for 48 hours. After two days of infection, the viral media was removed, and complete HEK293E medium supplemented with 2.05 μg/mL of puromycin was added to each well to select transduced cells for two days. Transduced HEK293E cells were then maintained on complete media supplemented with 2.05 μg/mL of puromycin for several passages, before being collected for analysis.

### Cell lysis and protein extraction from HEK293E cells

Cells were washed with 1 or 2 mL (12-well or 6-well dish) of ice-cold 1x PBS (Corning) prior to cell lysis. 100 or 200 μl (12-well or 6-well dish) of ice-cold lysis buffer (Thermo Scientific M-PER Mammalian Protein Extraction Reagent #78501) supplemented with Roche Complete Protease Inhibitor Cocktail (#11697498001) was added to each experimental well, on ice. Plates were shaken at 4°C for 10 minutes. Lysates were spun down at 16000g for 15 minutes at 4°C to remove insoluble material. Protein concentrations were determined using the BCA Protein Assay Kit (Pierce Thermo Scientific #23225). 4x Laemmli Buffer (Bio-Rad Laboratories #1610747) was added and samples were boiled at 100°C for 5 minutes.

### Western blotting

Equal amounts of protein (35-75μg of protein per well depending on experimental condition) were resolved on 10- or 15-well 4–20% Mini-PROTEAN TGX Stain-Free Protein Gels (Bio-Rad Laboratories #4568093 and #4568096) and transferred onto nitrocellulose membranes (BioTrace #66485). Membranes were blocked with either Odyssey Blocking Buffer (TBS) (Licor #927–50000) or soy milk with 20mM Tris-HCL and 0.1% Tween-20. Immunoblotting was performed using the following primary antibodies: mouse anti-C-ATXN2 (BD Biosciences #611378, 1:1000 dilution), rabbit anti-HA (Cell Signaling Technologies #3724S, 1:1000), mouse anti-1C2 (polyQ) (Millipore Sigma #MAB1574, 1:1000), rabbit anti-β-tubulin (Abcam #ab6046,1:10000), mouse anti-GAPDH (Santa Cruz Biotechnology #sc-47724, 1:5000), and mouse anti-β-actin (Santa Cruz Biotechnology #sc-47778, 1:10000). Corresponding red and green fluorescent secondary antibodies (Licor #926–68070, #926–32210, and #926–32211, 1:10000) were used for visualization with the Odyssey CLx Infrared Imaging System (LI-COR Biosciences). All Western blot membranes were imaged using the Automatic setting, which acquires images with no saturated pixels without user adjustments. Brightness and contrast of some images were adjusted to better visualize Western blot bands.

### Immunoprecipitation

#### Bead preparation

Protein A Sepharose 4 Fast Flow Beads (GE Healthcare #17-5280-01) were washed with 1mL of ice-cold 1x PBS and then blocked with 0.1% BSA in PBS for 1 hour while rotating at 4°C. Beads were washed twice with 1 mL ice-cold PBS and once with Isotonic Wash Buffer (20 mM Tris-HCl pH 7.5, 150mM NaCl, 0.1% NP-40). Beads were conjugated with the indicated antibodies or immunoglobulin (IgG) at a dilution of 1:125 in 1mL of Isotonic Wash Buffer for 3 hours at 4°C while rotating. Beads were then washed twice with 1mL of ice-cold Isotonic Wash Buffer.

#### Transfection and lysate preparation for immunoprecipitation

HEK293E cells were plated in 100mm^2^ dishes at a density of ~3,000,000 cells/dish and transfected as described above, but with 2.1μg of appropriate cDNA combined with 21ul of Mirus-LT1 Transfection Reagent (Mirus Bio #MIR 2300) and 1.75 mL of OptiMEM Reduced Serum Medium (Gibco #31985–070). 48 hours post-transfection, cells were lysed in 1.0 mL ice-cold lysis buffer supplemented with Roche protease inhibitor cocktail. Cell lysates were centrifuged at 16,000g at 4°C for 15 minutes and protein concentrations were determined by BCA protein assay. 200μL of lysate (equivalent to ~800 μg of cellular protein) was added to the antibody-conjugated beads and incubated while rotating at 4°C overnight.

#### Immunoprecipitation

Following overnight lysate incubation, beads were spun at 3000g at 4°C for 1 minute. Supernatant was removed and beads were washed 4 times with 1mL ice-cold Isotonic Wash Buffer. Recovered proteins were eluted in 30 μL of 1x Laemmli Buffer (diluted from 2x in cell lysis buffer), 5 minutes at room temperature, followed by a 5-minute incubation at 100°C. Beads recovered by centrifugation (3000xg, 5 min) and eluates were analyzed by immunoblotting, as described above.

### Western blot quantification and statistical analysis

Quantitative analysis was carried out using ImageStudio Software Version 5.2.5 (LI-COR Biosciences) on non-saturated Western blot bands. The signal intensity of each Western blot band was collected using the Western blot analysis setting on ImageStudio that subtracts the background signal of each lane from the signal of each band of interest. Band signal intensity values were not normalized to a loading control because the values calculated for quantitative analysis were ratios that corresponded to the signal intensity of an individual lane’s cleavage fragment band divided by the signal intensity of the total N-terminal signal recorded for both the full-length protein and corresponding N-terminal fragment in each lane. Note that while for some blots we adjusted brightness and contrast, this had no impact on quantification. Indeed, signal quantification is based on the original Western blot image capture (see above), and is thus independent of subsequent brightness and contrast adjustments.

Statistical analysis was performed with Prism 9 (GraphPad). Unpaired t-test was used to determine statistical significance when comparing two sets of measurements. One-way ANOVA followed by Dunnett’s multiple comparisons test was used for experiments with more than two groups. All error bars in graphs represent the standard error of the mean.

## Results

### ATXN2 undergoes N-terminal proteolysis in HEK293E cells

To determine whether ATXN2 can undergo N-terminal proteolytic cleavage, and whether an N-terminal polyQ-containing product might accumulate, we expressed full length ATXN2 proteins in HEK293E cells, using constructs that included the most 5’ of the two known potential ATG start codons [19] and an N-terminal HA tag, since there is currently no available N-terminally-directed anti-ATXN2 antibody (Fig 1A). We expressed both WT ATXN2 with a normal polyQ domain of 22 amino acids (ATG1-HA-ATXN2-22Q), and mutant ATXN2 with a pathogenic repeat of 39 glutamines (ATG1-HA-ATXN2-39Q). Extracts from these transfected cells were immunoblotted using both C-terminally-directed ATXN2 (C-ATXN2) and N-terminally-directed HA antibodies (Fig 1B, S2A and S2B Fig). The C-ATXN2 antibody detected two bands that could correspond to the full-length protein. The first band–with an apparent molecular weight of ~145 kDa–migrated similarly to a protein detected in cells transfected with an empty vector. This endogenous protein was ATXN2, since its level was reduced in six HEK293E cell lines, each expressing a different shRNA targeting the *ATXN2* mRNA (S1A and S1B Fig). GAPDH levels were unaffected in these cell lines, demonstrating the specificity of the shRNAs targeting *ATXN2* (S1A and S1C Fig). The second band detected with the C-ATXN2 antibody in cells expressing HA-tagged ATXN2 proteins unexpectedly migrated much more slowly than endogenous ATXN2, with an apparent molecular weight of ~180 kDa (Fig 1B, S2A and S2B Fig). Interestingly, the HA antibody detected the ~180 kDa isoform, but not the ~145 kDa band (Fig 1B, S2A and S2B Fig). Furthermore, there was a subtle, but reproducible difference in migration between the slower migrating bands observed with WT (22Q) and mutant (39Q) polyQ domains, while the migration of the ~145 kDa band was insensitive to polyQ length. These results suggested that the ~180 kDa band contains both the N-terminal and polyQ domains, while the ~145 kDa band does not contain the N terminus, possibly due to proteolytic cleavage. Consistent with this premise, we detected small HA-immunoreactive bands migrating at ~27 and 30 kDa in extracts from cells expressing WT and mutant ATXN2, respectively (Fig 1B, S2A and S2B Fig), suggesting that both normal and mutant ATXN2 proteins are subject to proteolysis. The difference in migration between the two fragments when probing for HA indicated that these fragments contain the polyQ domain and migrate differently due to the differential length of the polyQ domain.

We noted that an additional band sensitive to polyQ length was consistently detected at ~65–70 kDa with the HA antibody (S2A and S2B Fig). A ~115 kDa band was also observed with the C-terminal ATXN2 antibody in lysates expressing both HA-tagged or untagged ATG1-ATXN2 lysates, and the molecular weight of this band did not vary in response to changes in polyQ length (S2A and S2B Fig). Based on their apparent sizes, these two bands could be the product of a second proteolytic cleavage event. The signal strength of the ~115 kDa band varied across experiments as opposed to the more stable ~180 and 145 kDa bands. Additional bands, even more variable, could be seen, particularly when expression levels were high or signal boosted. For example, a weak band was detected around 20 kDa, which migrated more slowly when originating from mutant ATXN2 (S2A Fig). However, since the antibody we used recognizes the ATXN2 C-terminal domain and the polyQ is located in the N-terminal region (Fig 1A), this fragment is too small to contain the polyQ domain. It thus appears that the length of the polyQ repeat indirectly affects the cleavage site producing the 20 kDa fragment. We focused this study on the consistent proteolysis event that generates the N-terminal ~27 kDa and C-terminal ~145 kDa fragments.

To determine whether the ~27 kDa N-terminal fragment and the ~180 kDa ATXN2 isoform indeed contain the polyQ repeat, we used an anti-polyQ antibody (1C2) [32] that detects extended polyQ domains, but not shorter, WT polyQ repeats [33]. 1C2 detected a polyQ stretch in both the small HA-positive N-terminal fragment and the ~180 kDa isoform from mutant lysates, but not from WT lysates, as expected (Fig 1C). On the other hand, 1C2 did not detect the ~145kd band in mutant protein extracts. These results demonstrate that the N-terminal fragment and the ~180 kDa ATXN2 isoform contain the polyQ repeat, while the ~145kd fragment does not.

To investigate whether the N-terminal and C-terminal fragments of ATXN2 remain associated after cleavage, we expressed WT or mutant ATXN2 and performed immunoprecipitation with anti-HA antibodies. Full-length ATXN2 and the N-terminal fragment were efficiently immunoprecipitated with the HA antibody, but not the ~145 kDa C-terminal fragment (Fig 1D). These results suggested that upon proteolytic cleavage, the N-terminal fragment does not remain associated with the rest of the protein, and further validated that the ~145 kDa band lacks an N-terminus.

In conclusion, when overexpressed in HEK293E cells, ATXN2 undergoes N-terminal proteolytic cleavage. The slower migrating isoform is the full-length protein, while the ~145 kDa protein isoform is a cleavage product containing all known functional protein domains but lacking the polyQ domain. Finally, a small N-terminal fragment containing the polyQ domain accumulates. Note that since endogenous ATXN2 and the ~145 kDa product comigrate, we cannot exclude that transfected ATXN2 increased endogenous ATXN2 level, and that this contributed to the enhanced intensity of the ~145 kDa band in transfected cells.

### A 17 amino acid domain C-terminal of the polyQ domain is necessary and sufficient for N-terminal cleavage of ATXN2

We next sought to understand how ATXN2 undergoes proteolytic cleavage and leveraged a deletion mapping strategy to identify the key protein domain(s) promoting its cleavage. Since the results described above demonstrated that the cleavage occurred downstream of the polyQ domain, we first generated a construct encoding a protein fusion comprising 395 N-terminal amino acids of ATXN2 and EGFP, with a short linker sequence separating the two moieties (Fig 2A). This fusion protein was proteolytically processed and produced a ~27 kDa N-terminal cleavage fragment similar to that seen in WT ATXN2 lysates. This indicated that the C-terminal region of ATXN2 is not required for the N-terminal cleavage to occur (Fig 2B).

The N-terminal domain was then progressively deleted further in the C to N direction in increments of 40–41 amino acids (Fig 2A). Cleavage and generation of a ~27 kDa N-terminal fragment was observed with all fusion proteins, except with the fusion that included only the first 194 amino acids of ATXN2 (i.e. it contained only 7 amino acids after the polyQ, Fig 2C). Thus, a sequence immediately following the polyQ domain is needed for N-terminal proteolysis (Fig 2D). To refine mapping of the cleavage-promoting site, 10 amino acids were added back to produce an ATXN2-Nt-EGFP fusion that included the 17 amino acids immediately C-terminal of the polyQ domain (Fig 2A). Reintroducing these 10 amino acids to the N-terminal domain restored production of the ~27 kDa fragment, which indicated that the 17 amino acids following the polyQ are critical for ATXN2 cleavage (Fig 2E and 2F).

The above results point to a cleavage motif or perhaps a protease recognition site adjacent and downstream of the polyQ domain. To determine whether this short domain is necessary for cleavage, we internally deleted the 17 amino acids immediately downstream of the polyQ domain from the WT ATXN2 protein (Fig 3A). This deletion completely abolished cleavage, demonstrating the necessity of this short sequence for N-terminal proteolysis of full-length ATXN2 (Fig 3B and 3C). Interestingly, elements of this unique sequence are perfectly conserved in mammals from mice to humans regardless of whether ATXN2 contains a polyQ domain or not (Fig 3D). The amino acid sequences flanking the conserved sequences are also repetitive in nature and similar in terms of amino acid content, suggesting that this sequence may play an important role in ATXN2 function or metabolism (Fig 3D).

To test whether the domain C-terminally adjacent to the polyQ is sufficient for cleavage, we inserted 7 to 90 amino acids of this region between an N-terminal EGFP and a C-terminal mCherry (Fig 4A). All fusion proteins with 90 to 15 amino acids underwent specific proteolysis that cleaved EGFP from mCherry, while the fusion with only 7 ATXN2 amino acids (residues 188–194) produced very low levels of EGFP fragment at ~27 kDa (Fig 4B and 4C). Efficiency of cleavage was statistically significantly reduced when comparing the shortest fusion protein with the two longest, but statistical significance was not reached between the shortest and intermediate length fusions, as overall cleavage efficiency was quite variable across experiments. Importantly however, in all 6 experiments, the fusion protein with only 7 ATXN2 amino acids clearly exhibited the lowest cleavage efficiency (P<10^−5^ to have occurred by chance). These results thus illustrate the importance and sufficiency of the first 17 amino acids following the polyQ domain for ATXN2 cleavage. We also noted a trend for the two longer fusions being cleaved more efficiently than those with 15–60 amino acids (Fig 4C). Our results thus show that the first 17 amino acids following the polyQ are necessary and sufficient for ATXN2 cleavage, but a broader region might be needed for optimal cleavage efficiency.

### ATXN2 produced from the second ATG start codon can also undergo N-terminal proteolysis

The *ATXN2* gene produces multiple mRNA isoforms, but it is usually depicted as encoding a 1313 amino acid protein starting from ATG1, that includes 165 amino acids N-terminal of the polyQ domain [1, 10, 15, 20, 34–36] However, evidence suggests that the main translational start codon might be located just a few codons upstream of those encoding ATXN2’s polyQ domain [19]. This second start codon (ATG2) is predicted to produce a significantly shorter (124.4 kDa) protein than the first (ATG1, 140.3 kDa). To determine whether this shorter ATXN2 isoform also undergoes proteolytic cleavage, we generated HA-tagged constructs that included only ATG2 (Fig 5A). As with ATG1-containing constructs, HA-tagged ATG2-ATXN2 constructs produced two bands recognized by the C-ATXN2 antibody, with the upper band’s mobility dependent on polyQ length, while the faster migrating band was insensitive to polyQ length (Fig 5B, S2C, S3A Figs). The mobility of these two bands was reproducibly faster than the mobility of the two similar bands observed with ATG1 constructs, consistent with a shorter protein. This was expected for the slow-migrating band since it should correspond to the uncleaved ATG2-ATXN2 product. However, if the fast-migrating fragment was generated from N-terminal cleavage occurring at the same site as with the full-length (ATG1) ATXN2, gel mobility of the fast-migrating fragment should have been unchanged. HA staining demonstrated that the slow-migrating band observed with ATG2 contained the N-terminus, while the lower did not (Fig 5B). Thus, the shorter ATXN2 isoform produced from ATG2 also undergoes N-terminal proteolytic cleavage, but probably at a different site than ATG1-ATXN2. We noticed that the difference in size between the two bands detected with C-ATXN2 was clearly smaller than the size difference observed with ATG1-HA-ATXN2 (Fig 5B), which suggests that the resulting N-terminal ATG2 fragment is significantly smaller than the N-terminal ATG1 fragment, probably less than 15 kDa. We were not able to detect this fragment on our gels, either because it was too small, or had a short half-life.

We considered the possibility that the N-terminal HA tag may be influencing N-terminal cleavage behavior, especially when comparing the ATG1 and ATG2 isoforms of ATXN2. Indeed, ATG1-ATXN2 contains a much longer N-terminal sequence preceding the polyQ domain, while ATG2-ATXN2 only has 5 amino acids in the sequence prior to the polyQ tract. We thus generated untagged ATG2-ATXN2 constructs with both WT and mutant polyQ domains to compare their cleavage pattern against the untagged ATG1-ATXN2 constructs (Fig 5C). We found that both untagged ATG1-ATXN2 and ATG2-ATXN2 underwent N-terminal cleavage, as revealed by probing Western Blots with the C-terminal ATXN2 antibody (Fig 5D, S2D and S2E Fig). Additionally, even after removing the HA tag, the migration difference of the faster migrating C-terminal cleavage products bands was reproducibly observed when comparing ATG1 and ATG2 lysates (Fig 5D, S3B Fig). Thus, the presence of an HA tag had no impact on N-terminal proteolysis of ATG1-ATXN2 and ATG2-ATXN2.

Since the fast-migrating band corresponding to the ATG2-ATXN2 C-terminal cleavage product migrates faster than that of ATG1-ATXN2, N-terminal proteolysis likely occurs at different sites in the long and short ATXN2 isoforms. We therefore deleted the 17 amino acids we found to be necessary for cleavage of full-length (ATG1) ATXN2 from WT ATG2-ATXN2 (Fig 6A). HA signal was detected at ~160 kDa from extracts expressing the protein containing the deletion, while the faster migrating band was only detected by the C-terminal antibody, Thus, the 17-amino acid deletion did not abolish ATG2-ATXN2 cleavage (Fig 6B). In summary, we conclude that both full-length (ATG1) and the short (ATG2) ATXN2 isoforms undergo N-terminal proteolytic cleavage in HEK293 cells, but proteolysis of these two isoforms is dependent on distinct cleavage-promoting sites.

## Discussion

Several polyQ-containing proteins implicated in neurodegeneration are the target of N-terminal proteolysis, which produces small pathological polyQ fragments that accumulate in diseased brains and aggregate [18]. Interestingly, polyQ containing ATXN2 fragments have been observed in a brain extract from a deceased patient that carried a 41Q repeat [29]. However, N-terminal ATXN2 proteolysis has received little attention since, perhaps because nuclear inclusion body formation with elongated polyQ ATXN2 did not appear necessary for disease progression [29]. Our study establishes that when overexpressed in HEK293E cells, ATXN2 can indeed be targeted by such proteolytic events.

Our results are reminiscent of those obtained with the aforementioned SCA2 brain extract [29]: as in our study, a small polyQ-containing fragment was observed with the 1C2 antibody that recognizes elongated polyQ stretches, as well as a large, likely full-length protein migrating at about 180kd. A ~145 kDa protein was also observed in both the patient and a healthy individual, which was not recognized by the 1C2 antibody and thus did not contain the extended polyQ domain. This apparent molecular weight of ~145 kDa is actually the commonly reported one for ATXN2, and fits reasonably well with its predicted full length of 1313 amino acids [1, 10, 15, 17, 20, 34–36]. However, we show here that full-length ATXN2 actually migrates more slowly than predicted, with an apparent molecular weight of ~ 180 kDa. Thus, the commonly observed ~145kDa bands should correspond to shorter isoform(s), even though ATXN2 is usually represented as a 1313 amino acid long protein [1, 10, 15, 20, 34–36]. Our results show that N-terminal protein cleavage of full-length ATXN2 can produce a shorter ~145 kDa isoform. In addition, the use of the second alternative start codon (ATG2) [19], possibly combined with alternative splicing [20], could also result in isoforms of similar sizes. Either mechanism could explain the presence of a ~145 kDA band that is not recognized by the 1C2 antibody in both a healthy individual and patient carrying both a normal and a pathogenic *Atxn2* allele [29]: that band could contain a cleaved product and/or a short isoform produced from a wild-type allele. We note that in a very recent study, a slow-migrating ATXN2 isoform was also observed after transient transfection in Hela, COS7 and HEK293 cells [20], that is probably identical to the ~180 kDa full-length ATXN2 band we observed, but its nature was not determined in that work. Thus, ATXN2 N-terminal cleavage likely occurs in multiple cultured cell lines, and this should be taken into careful consideration by future investigators, particularly when using N-terminal ATXN2 fusion proteins.

Our results show that ATXN2 can be cleaved at a very specific site. Interestingly, the N-terminus is, itself, a determinant of ATXN2 proteolytic cleavage. Indeed, we identified a 17 amino acid sequence that is necessary and sufficient for proteolysis of full-length ATXN2 generated from ATG1, but this sequence is dispensable for cleavage of the shorter ATG2-dependent ATXN2 isoform. This suggests that ATXN2’s N-terminal conformation and possibly its incorporation into multi-protein complexes determine how efficiently and where the currently unknown ATXN2 protease is able to cleave ATXN2. It should be acknowledged that while we favor the idea that the 17 amino acid sequence adjacent to the polyQ stretch is the site of ATXN2 cleavage, we cannot exclude that this site is used as a recognition site for the ATXN2 protease that then cleaves ATXN2 at a nearby site.

It is important to note that in fibroblastic cell lines derived from SCA2 patients, or in cell lines in which a long isoform allele was engineered, migration of the main ATXN2 band was observed to be dependent on polyQ length [37]. This indicates that in these cells, which do not overexpress ATXN2, this protein is not cleaved N-terminally, or very inefficiently. It is therefore possible that cleavage is more likely if ATXN2 accumulates excessively or behaves abnormally, because of overexpression or pathology. Future work is needed to determine *in vivo* in which tissues and under which conditions ATXN2 can be cleaved N-terminally. The functional consequences of the protein cleavage would also be interesting to study. Of the known ATXN2 domains, only the polyQ domain would be missing after cleavage. We would thus predict that the cleaved product is functional, but it might have reduced activity due to lower affinity for its binding partners since polyQ stretches can impact protein interactions [38, 39]. This could have important impact on expression of ATXN2 target genes [9].

As mentioned above, the existence of small polyQ-containing N-terminal fragments in at least one SCA2 patient is intriguing [29]. Designating ATXN2 as a target of N-terminal proteolysis could open new therapeutic avenues for ATXN2-related neurodegeneration. There are several examples of how either inhibiting N-terminal cleavage of polyQ proteins or targeting their N-terminal cleavage fragments for degradation ameliorate neurodegenerative disease pathology. For example, mutating the caspase 7 cleavage site in ATXN7 produces a cleavage-resistant form of ATXN7, and the disease pathology of SCA7 model mice improves despite an expanded polyQ tract [23]. Mice expressing a mutant form of Huntingtin that cannot be cleaved by caspase-6 also fail to develop neurodegenerative phenotypes despite having an expanded-polyQ form of the gene [28]. Inhibiting calpain cleavage of ATXN3 improved disease phenotype in SCA3 mice as well [25]. Additionally, reducing accumulation of N-terminal cleavage fragments of neurodegenerative polyQ proteins improves phenotypes associated with disease progression in the context of SCA7 [40] and Huntington’s disease [41]. In the context of disease, ATXN2 is known to accumulate as shown with N-terminal 1C2 [42, 43] and C-terminal ATXN2 staining [5]. Interestingly, cell culture studies have shown that a ~500 amino acid N-terminal fragment of ATXN2 fused to GFP is phosphorylated via Cdk5-p25 and thus targeted for degradation via the proteasome [21]. The E3 ubiquitin ligase Parkin might be implicated in this degradation [34]. Intriguingly, most putative Cdk5 phosphorylation sites in this ~500 amino acid fragment are located N-terminal of the polyQ. Thus, Cdk5 could be a pharmacological target to promote N-terminal cleavage product clearance. It would therefore be important to determine whether N-terminal fragment accumulation is a factor in the progression of neurodegenerative diseases caused by ATXN2.

## Supporting information

S1 Fig The endogenous ~145 kDA band corresponds to ATXN2. **A.** Western blot for ATXN2 (C-ATXN2, red), tubulin (blue), and GAPDH (red) in HEK293E cells stably transfected with empty vector (pGIPZ), a non-silencing shRNA (NS), an shRNA targeting GAPDH, or six different shRNAs targeting ATXN2. A Western blot representative of four (GAPDH) or five (ATXN2) independent experiments is shown. **B and C.** Quantification of ATXN2 (B) and GAPDH (C) levels, normalized using tubulin signal. ATXN2 or GAPDH levels in pGIPZ cells were set to 1. Statistical analysis performed with one-way ANOVA with Dunnett’s multiple comparisons test. **P < 0.01, ****P < 0.0001.
(TIF)

S2 Fig ATG1 and ATG2-ATXN2 protein products in HEK293 cells. **A.** Complete view of a Western blot for ATG1-HA-ATXN2-22Q and ATG1-HA-ATXN2-39Q. HEK293E cells were transfected with either ATG1-HA-ATXN2-22Q or ATG1-HA- ATXN2-39Q and cell lysates were analyzed by immunoblotting. Blue: Anti-HA, Red: Anti-C-ATXN2, Magenta: co-localized bands. FL = full-length ATG1-HA-ATXN2 protein; CT = C-terminal fragment; NT = N-terminal fragment. A Western blot representative of four independent experiments is shown. **B.** Additional complete view of Western blot for ATG1-HA-ATXN2-22Q and ATG1-HA-ATXN2-39Q with β-Actin loading control. HEK293E cells were transfected with either ATG1-HA-ATXN2-22Q or ATG1-HA- ATXN2-39Q and cell lysates were analyzed by immunoblotting. Blue: Anti-HA, Red: Anti-C-ATXN2 or β-Actin, Magenta: co-localized bands. A Western blot representative of four independent experiments is shown. **C.** Complete view of Western blot for ATG2-HA-ATXN2-22Q and ATG2-HA-ATXN2-40Q (also shown in Fig 5). HEK293E cells were transfected with plasmids shown in Fig 5A, and cell lysates were analyzed by immunoblotting. Blue: Anti-HA; Red: Anti-C-ATXN2 or β-Actin. A Western blot representative of three experiments is shown. **D.** Western blot for untagged ATG1-ATXN2- 22Q and ATG1-ATXN2-39Q. HEK293E cells were transfected with plasmids shown in Fig 5C, and cell lysates were analyzed by immunoblotting. The top half of the membrane was probed with C-ATXN2 antibody while the bottom half was probed with β-Actin. A Western blot representative of three independent experiments is shown. **E.** Western blot for untagged ATG2-ATXN2- 22Q and ATG2-ATXN2-37Q. HEK293E cells were transfected with plasmids shown in Fig 5C, and cell lysates were analyzed by immunoblotting. The top half of the membrane was probed with C-ATXN2 antibody while the bottom half was probed with β-Actin. A Western blot representative of three experiments is shown.
(TIF)

S3 Fig ATG1 and ATG2 C-terminal fragments migrate differently. **A.** An additional representative blot illustrating the differing molecular weights of the HA-ATG1 vs. HA-ATG2 C-terminal fragments. HEK293E cells were transfected with plasmids shown in Fig 5A, and cell lysates were analyzed by immunoblotting. Red: Anti-C-ATXN2 or β-Actin, Blue: Anti-HA, Magenta: co-localized bands. FL = full-length ATG1-HA-ATXN2 and ATG2-HA-ATXN2 protein; CT = C-terminal fragment. * signs indicate the slower migration of mutant full-length ATXN2 proteins; Arrowheads and dotted lines, positioned in the middle of bands, emphasize migration difference between ATG1-HA-ATXN2 and ATG2-HA-ATXN2 C-terminal fragments. Flat arrowheads: ATG1-HA-ATXN2 C-terminal fragment; Curved arrowheads: ATG2-HA-ATXN2 C-terminal fragment. A Western blot representative of three independent experiments is shown. **B.** An additional representative blot illustrating the differing molecular weights of the ATG1 vs. ATG2 C-terminal fragments. HEK293E cells were transfected with plasmids shown in Fig 5C, and cell lysates were analyzed by immunoblotting. Red: Anti-C-ATXN2 or β-Actin, FL = full-length ATG1-ATXN2 and ATG2-ATXN2 protein; CT = C-terminal fragment. * signs indicate the slower migration of mutant full-length ATXN2 proteins; Arrowheads and dotted lines, positioned in the middle of bands, emphasize migration difference between ATG1-ATXN2 and ATG2-ATXN2 C-terminal fragments. Flat arrowheads: ATG1-ATXN2 C-terminal fragment; Curved arrowheads: ATG2-ATXN2 C-terminal fragment. A Western blot representative of three independent experiments is shown.
(TIF)

S1 Raw images (PDF)

## References

[1] Lastres-Becker I, Rub U, Auburger G. Spinocerebellar ataxia 2 (SCA2). Cerebellum. 2008;7(2):115–24. Epub 2008/04/18. doi: 10.1007/s12311-008-0019-y . 18418684

[2] Nechiporuk A, Lopes-Cendes I, Nechiporuk T, Starkman S, Andermann E, Rouleau GA, et al. Genetic mapping of the spinocerebellar ataxia type 2 gene on human chromosome 12. Neurology. 1996;46(6):1731–5. Epub 1996/06/01. doi: 10.1212/wnl.46.6.1731 . 8649579

[3] Sanpei K, Takano H, Igarashi S, Sato T, Oyake M, Sasaki H, et al. Identification of the spinocerebellar ataxia type 2 gene using a direct identification of repeat expansion and cloning technique, DIRECT. Nat Genet. 1996;14(3):277–84. Epub 1996/11/01. doi: 10.1038/ng1196-277 . 8896556

[4] Pulst SM, Nechiporuk A, Nechiporuk T, Gispert S, Chen XN, Lopes-Cendes I, et al. Moderate expansion of a normally biallelic trinucleotide repeat in spinocerebellar ataxia type 2. Nat Genet. 1996;14(3):269–76. Epub 1996/11/01. doi: 10.1038/ng1196-269 . 8896555

[5] Elden AC, Kim HJ, Hart MP, Chen-Plotkin AS, Johnson BS, Fang X, et al. Ataxin-2 intermediate-length polyglutamine expansions are associated with increased risk for ALS. Nature. 2010;466(7310):1069–75. Epub 2010/08/27. doi: 10.1038/nature09320 . 20740007 PMC2965417

[6] Wang JL, Xiao B, Cui XX, Guo JF, Lei LF, Song XW, et al. Analysis of SCA2 and SCA3/MJD repeats in Parkinson’s disease in mainland China: genetic, clinical, and positron emission tomography findings. Mov Disord. 2009;24(13):2007–11. Epub 2009/08/13. doi: 10.1002/mds.22727 . 19672991

[7] Modoni A, Contarino MF, Bentivoglio AR, Tabolacci E, Santoro M, Calcagni ML, et al. Prevalence of spinocerebellar ataxia type 2 mutation among Italian Parkinsonian patients. Mov Disord. 2007;22(3):324–7. Epub 2006/12/07. doi: 10.1002/mds.21228 . 17149720

[8] McCann C, Holohan EE, Das S, Dervan A, Larkin A, Lee JA, et al. The Ataxin-2 protein is required for microRNA function and synapse-specific long-term olfactory habituation. Proc Natl Acad Sci U S A. 2011;108(36):E655–62. Epub 2011/07/29. doi: 10.1073/pnas.1107198108 . 21795609 PMC3169144

[9] Yokoshi M, Li Q, Yamamoto M, Okada H, Suzuki Y, Kawahara Y. Direct binding of Ataxin-2 to distinct elements in 3’ UTRs promotes mRNA stability and protein expression. Mol Cell. 2014;55(2):186–98. Epub 2014/06/24. doi: 10.1016/j.molcel.2014.05.022 . 24954906

[10] Inagaki H, Hosoda N, Tsuiji H, Hoshino SI. Direct evidence that Ataxin-2 is a translational activator mediating cytoplasmic polyadenylation. J Biol Chem. 2020;295(47):15810–25. Epub 2020/09/30. doi: 10.1074/jbc.RA120.013835 . 32989052 PMC7681009

[11] Lim C, Allada R. ATAXIN-2 activates PERIOD translation to sustain circadian rhythms in Drosophila. Science. 2013;340(6134):875–9. Epub 2013/05/21. doi: 10.1126/science.1234785 . 23687047

[12] Zhang Y, Ling J, Yuan C, Dubruille R, Emery P. A role for Drosophila ATX2 in activation of PER translation and circadian behavior. Science. 2013;340(6134):879–82. Epub 2013/05/21. doi: 10.1126/science.1234746 . 23687048 PMC4078874

[13] Ciosk R, DePalma M, Priess JR. ATX-2, the C. elegans ortholog of ataxin 2, functions in translational regulation in the germline. Development. 2004;131(19):4831–41. Epub 2004/09/03. doi: 10.1242/dev.01352 . 15342467

[14] Lastres-Becker I, Nonis D, Eich F, Klinkenberg M, Gorospe M, Kotter P, et al. Mammalian ataxin-2 modulates translation control at the pre-initiation complex via PI3K/mTOR and is induced by starvation. Biochim Biophys Acta. 2016;1862(9):1558–69. Epub 2016/06/01. doi: 10.1016/j.bbadis.2016.05.017 . 27240544 PMC4967000

[15] Bakthavachalu B, Huelsmeier J, Sudhakaran IP, Hillebrand J, Singh A, Petrauskas A, et al. RNP-Granule Assembly via Ataxin-2 Disordered Domains Is Required for Long-Term Memory and Neurodegeneration. Neuron. 2018;98(4):754–66 e4. Epub 2018/05/18. doi: 10.1016/j.neuron.2018.04.032 . 29772202

[16] Kaehler C, Isensee J, Nonhoff U, Terrey M, Hucho T, Lehrach H, et al. Ataxin-2-like is a regulator of stress granules and processing bodies. PLoS One. 2012;7(11):e50134. Epub 2012/12/05. doi: 10.1371/journal.pone.0050134 . 23209657 PMC3507954

[17] Nonhoff U, Ralser M, Welzel F, Piccini I, Balzereit D, Yaspo ML, et al. Ataxin-2 interacts with the DEAD/H-box RNA helicase DDX6 and interferes with P-bodies and stress granules. Mol Biol Cell. 2007;18(4):1385–96. Epub 2007/03/30. doi: 10.1091/mbc.e06-12-1120 . 17392519 PMC1838996

[18] Shao J, Diamond MI. Polyglutamine diseases: emerging concepts in pathogenesis and therapy. Hum Mol Genet. 2007;16 Spec No. 2:R115–23. Epub 2007/10/04. doi: 10.1093/hmg/ddm213 . 17911155

[19] Scoles DR, Pflieger LT, Thai KK, Hansen ST, Dansithong W, Pulst SM. ETS1 regulates the expression of ATXN2. Hum Mol Genet. 2012;21(23):5048–65. Epub 2012/08/24. doi: 10.1093/hmg/dds349 . 22914732 PMC3490512

[20] Lastres-Becker I, Nonis D, Nowock J, Auburger G. New alternative splicing variants of the ATXN2 transcript. Neurol Res Pract. 2019;1:22. Epub 2019/07/03. doi: 10.1186/s42466-019-0025-1 . 33324888 PMC7650068

[21] Asada A, Yamazaki R, Kino Y, Saito T, Kimura T, Miyake M, et al. Cyclin-dependent kinase 5 phosphorylates and induces the degradation of ataxin-2. Neurosci Lett. 2014;563:112–7. Epub 2014/02/04. doi: 10.1016/j.neulet.2014.01.046 . 24486837

[22] Goldberg YP, Nicholson DW, Rasper DM, Kalchman MA, Koide HB, Graham RK, et al. Cleavage of huntingtin by apopain, a proapoptotic cysteine protease, is modulated by the polyglutamine tract. Nat Genet. 1996;13(4):442–9. Epub 1996/08/01. doi: 10.1038/ng0896-442 . 8696339

[23] Guyenet SJ, Mookerjee SS, Lin A, Custer SK, Chen SF, Sopher BL, et al. Proteolytic cleavage of ataxin-7 promotes SCA7 retinal degeneration and neurological dysfunction. Hum Mol Genet. 2015;24(14):3908–17. Epub 2015/04/11. doi: 10.1093/hmg/ddv121 . 25859008 PMC4476441

[24] Hubener J, Weber JJ, Richter C, Honold L, Weiss A, Murad F, et al. Calpain-mediated ataxin-3 cleavage in the molecular pathogenesis of spinocerebellar ataxia type 3 (SCA3). Hum Mol Genet. 2013;22(3):508–18. Epub 2012/10/27. doi: 10.1093/hmg/dds449 . 23100324

[25] Simoes AT, Goncalves N, Nobre RJ, Duarte CB, Pereira de Almeida L. Calpain inhibition reduces ataxin-3 cleavage alleviating neuropathology and motor impairments in mouse models of Machado-Joseph disease. Hum Mol Genet. 2014;23(18):4932–44. Epub 2014/05/13. doi: 10.1093/hmg/ddu209 . 24817574

[26] Miller JP, Holcomb J, Al-Ramahi I, de Haro M, Gafni J, Zhang N, et al. Matrix metalloproteinases are modifiers of huntingtin proteolysis and toxicity in Huntington’s disease. Neuron. 2010;67(2):199–212. Epub 2010/07/31. doi: 10.1016/j.neuron.2010.06.021 . 20670829 PMC3098887

[27] Mangiarini L, Sathasivam K, Seller M, Cozens B, Harper A, Hetherington C, et al. Exon 1 of the HD gene with an expanded CAG repeat is sufficient to cause a progressive neurological phenotype in transgenic mice. Cell. 1996;87(3):493–506. Epub 1996/11/01. doi: 10.1016/s0092-8674(00)81369-0 . 8898202

[28] Graham RK, Deng Y, Slow EJ, Haigh B, Bissada N, Lu G, et al. Cleavage at the caspase-6 site is required for neuronal dysfunction and degeneration due to mutant huntingtin. Cell. 2006;125(6):1179–91. Epub 2006/06/17. doi: 10.1016/j.cell.2006.04.026 . 16777606

[29] Huynh DP, Figueroa K, Hoang N, Pulst SM. Nuclear localization or inclusion body formation of ataxin-2 are not necessary for SCA2 pathogenesis in mouse or human. Nat Genet. 2000;26(1):44–50. Epub 2000/09/06. doi: 10.1038/79162 . 10973246

[30] Smith BN, Newhouse S, Shatunov A, Vance C, Topp S, Johnson L, et al. The C9ORF72 expansion mutation is a common cause of ALS+/-FTD in Europe and has a single founder. Eur J Hum Genet. 2013;21(1):102–8. Epub 2012/06/14. doi: 10.1038/ejhg.2012.98 . 22692064 PMC3522204

[31] Imbert G, Saudou F, Yvert G, Devys D, Trottier Y, Garnier JM, et al. Cloning of the gene for spinocerebellar ataxia 2 reveals a locus with high sensitivity to expanded CAG/glutamine repeats. Nat Genet. 1996;14(3):285–91. Epub 1996/11/01. doi: 10.1038/ng1196-285 . 8896557

[32] Lescure A, Lutz Y, Eberhard D, Jacq X, Krol A, Grummt I, et al. The N-terminal domain of the human TATA-binding protein plays a role in transcription from TATA-containing RNA polymerase II and III promoters. EMBO J. 1994;13(5):1166–75. Epub 1994/03/01. doi: 10.1002/j.1460-2075.1994.tb06366.x . 7510635 PMC394926

[33] Scoles DR, Ho MH, Dansithong W, Pflieger LT, Petersen LW, Thai KK, et al. Repeat Associated Non-AUG Translation (RAN Translation) Dependent on Sequence Downstream of the ATXN2 CAG Repeat. PLoS One. 2015;10(6):e0128769. Epub 2015/06/19. doi: 10.1371/journal.pone.0128769 . 26086378 PMC4472729

[34] Huynh DP, Nguyen DT, Pulst-Korenberg JB, Brice A, Pulst SM. Parkin is an E3 ubiquitin-ligase for normal and mutant ataxin-2 and prevents ataxin-2-induced cell death. Exp Neurol. 2007;203(2):531–41. Epub 2006/11/14. doi: 10.1016/j.expneurol.2006.09.009 . 17097639 PMC2788988

[35] Ostrowski LA, Hall AC, Mekhail K. Ataxin-2: From RNA Control to Human Health and Disease. Genes (Basel). 2017;8(6). Epub 2017/06/08. doi: 10.3390/genes8060157 . 28587229 PMC5485521

[36] Albrecht M, Golatta M, Wullner U, Lengauer T. Structural and functional analysis of ataxin-2 and ataxin-3. Eur J Biochem. 2004;271(15):3155–70. Epub 2004/07/22. doi: 10.1111/j.1432-1033.2004.04245.x . 15265035

[37] Paul S, Dansithong W, Figueroa KP, Scoles DR, Pulst SM. Staufen1 links RNA stress granules and autophagy in a model of neurodegeneration. Nat Commun. 2018;9(1):3648. Epub 2018/09/09. doi: 10.1038/s41467-018-06041-3 . 30194296 PMC6128856

[38] Petrakis S, Schaefer MH, Wanker EE, Andrade-Navarro MA. Aggregation of polyQ-extended proteins is promoted by interaction with their natural coiled-coil partners. Bioessays. 2013;35(6):503–7. Epub 2013/03/14. doi: 10.1002/bies.201300001 . 23483542 PMC3674527

[39] Schaefer MH, Wanker EE, Andrade-Navarro MA. Evolution and function of CAG/polyglutamine repeats in protein-protein interaction networks. Nucleic Acids Res. 2012;40(10):4273–87. Epub 2012/01/31. doi: 10.1093/nar/gks011 . 22287626 PMC3378862

[40] Mookerjee S, Papanikolaou T, Guyenet SJ, Sampath V, Lin A, Vitelli C, et al. Posttranslational modification of ataxin-7 at lysine 257 prevents autophagy-mediated turnover of an N-terminal caspase-7 cleavage fragment. J Neurosci. 2009;29(48):15134–44. Epub 2009/12/04. doi: 10.1523/JNEUROSCI.4720-09.2009 . 19955365 PMC2907146

[41] Warby SC, Doty CN, Graham RK, Shively J, Singaraja RR, Hayden MR. Phosphorylation of huntingtin reduces the accumulation of its nuclear fragments. Mol Cell Neurosci. 2009;40(2):121–7. Epub 2008/11/11. doi: 10.1016/j.mcn.2008.09.007 . 18992820

[42] Koyano S, Yagishita S, Kuroiwa Y, Tanaka F, Uchihara T. Neuropathological staging of spinocerebellar ataxia type 2 by semiquantitative 1C2-positive neuron typing. Nuclear translocation of cytoplasmic 1C2 underlies disease progression of spinocerebellar ataxia type 2. Brain Pathol. 2014;24(6):599–606. Epub 2014/03/29. doi: 10.1111/bpa.12146 . 24674145 PMC8028922

[43] Seidel K, Siswanto S, Fredrich M, Bouzrou M, den Dunnen WFA, Ozerden I, et al. On the distribution of intranuclear and cytoplasmic aggregates in the brainstem of patients with spinocerebellar ataxia type 2 and 3. Brain Pathol. 2017;27(3):345–55. Epub 2016/07/06. doi: 10.1111/bpa.12412 . 27377427 PMC8028910

