# Supplementary figures and images for: ATXN2 is a target of N-terminal proteolysis
Source: PLoS One. 2023 Dec 21;18(12):e0296085. doi: 10.1371/journal.pone.0296085 (PMC10735043; doi:10.1371/journal.pone.0296085)

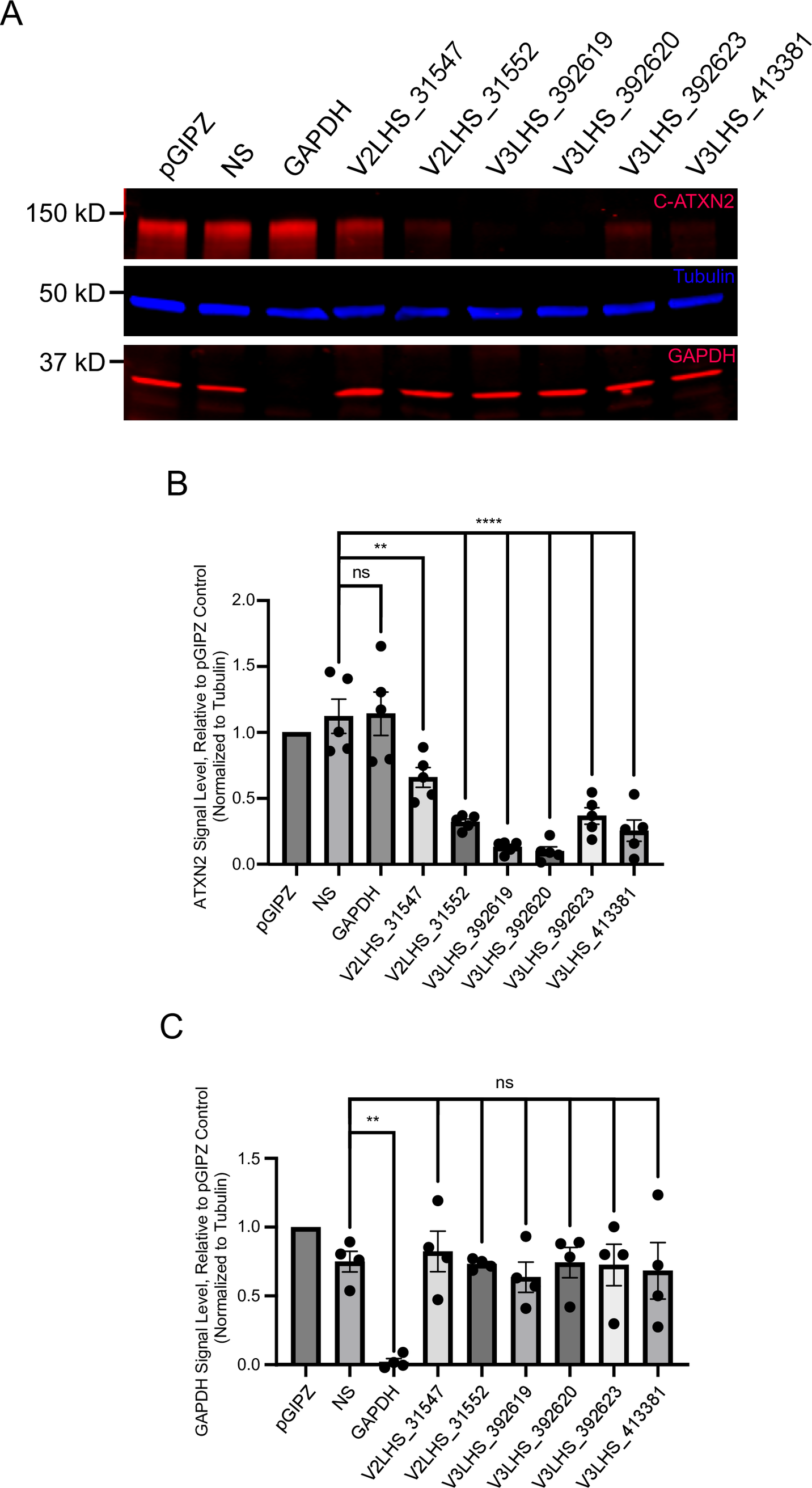

Supplement: S1 Fig — A. Western blot for ATXN2 (C-ATXN2, red), tubulin (blue), and GAPDH (red) in HEK293E cells stably transfected with empty vector (pGIPZ), a non-silencing shRNA (NS), an shRNA targeting GAPDH, or six different shRNAs targeting ATXN2. A Western blot representative of four (GAPDH) or five (ATXN2) independent experiments is shown. B and C. Quantification of ATXN2 (B) and GAPDH (C) levels, normalized using tubulin signal. ATXN2 or GAPDH levels in pGIPZ cells were set to 1. Statistical analysis performed with one-way ANOVA with Dunnett’s multiple comparisons test. **P < 0.01, ****P < 0.0001. (TIF) [file pone.0296085.s001.tif]

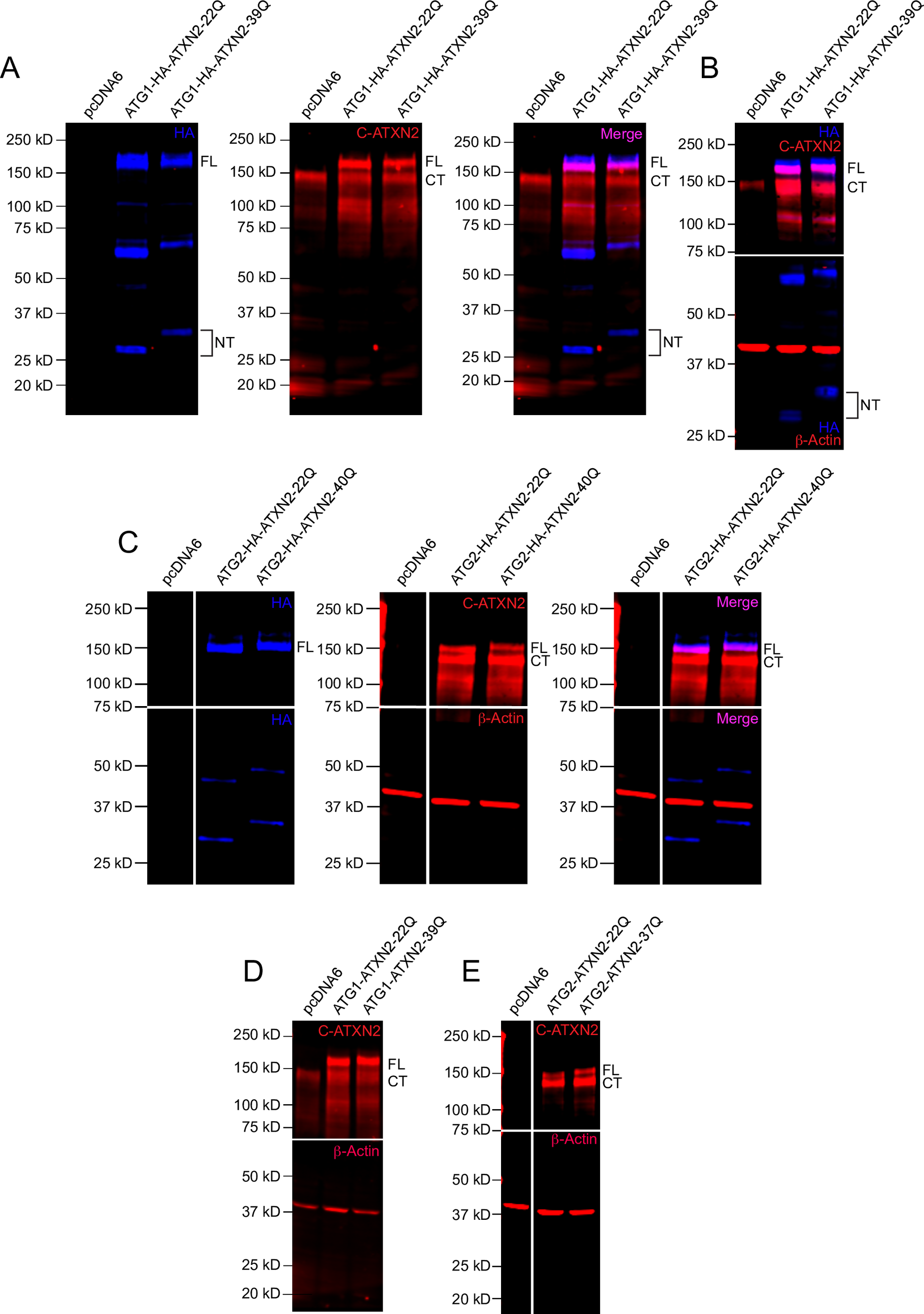

Supplement: S2 Fig — A. Complete view of a Western blot for ATG1-HA-ATXN2-22Q and ATG1-HA-ATXN2-39Q. HEK293E cells were transfected with either ATG1-HA-ATXN2-22Q or ATG1-HA- ATXN2-39Q and cell lysates were analyzed by immunoblotting. Blue: Anti-HA, Red: Anti-C-ATXN2, Magenta: co-localized bands. FL = full-length ATG1-HA-ATXN2 protein; CT = C-terminal fragment; NT = N-terminal fragment. A Western blot representative of four independent experiments is shown. B. Additional complete view of Western blot for ATG1-HA-ATXN2-22Q and ATG1-HA-ATXN2-39Q with β-Actin loading control. HEK293E cells were transfected with either ATG1-HA-ATXN2-22Q or ATG1-HA- ATXN2-39Q and cell lysates were analyzed by immunoblotting. Blue: Anti-HA, Red: Anti-C-ATXN2 or β-Actin, Magenta: co-localized bands. A Western blot representative of four independent experiments is shown. C. Complete view of Western blot for ATG2-HA-ATXN2-22Q and ATG2-HA-ATXN2-40Q (also shown in Fig 5). HEK293E cells were transfected with plasmids shown in Fig 5A, and cell lysates were analyzed by immunoblotting. Blue: Anti-HA; Red: Anti-C-ATXN2 or β-Actin. A Western blot representative of three experiments is shown. D. Western blot for untagged ATG1-ATXN2- 22Q and ATG1-ATXN2-39Q. HEK293E cells were transfected with plasmids shown in Fig 5C, and cell lysates were analyzed by immunoblotting. The top half of the membrane was probed with C-ATXN2 antibody while the bottom half was probed with β-Actin. A Western blot representative of three independent experiments is shown. E. Western blot for untagged ATG2-ATXN2- 22Q and ATG2-ATXN2-37Q. HEK293E cells were transfected with plasmids shown in Fig 5C, and cell lysates were analyzed by immunoblotting. The top half of the membrane was probed with C-ATXN2 antibody while the bottom half was probed with β-Actin. A Western blot representative of three experiments is shown. (TIF) [file pone.0296085.s002.tif]

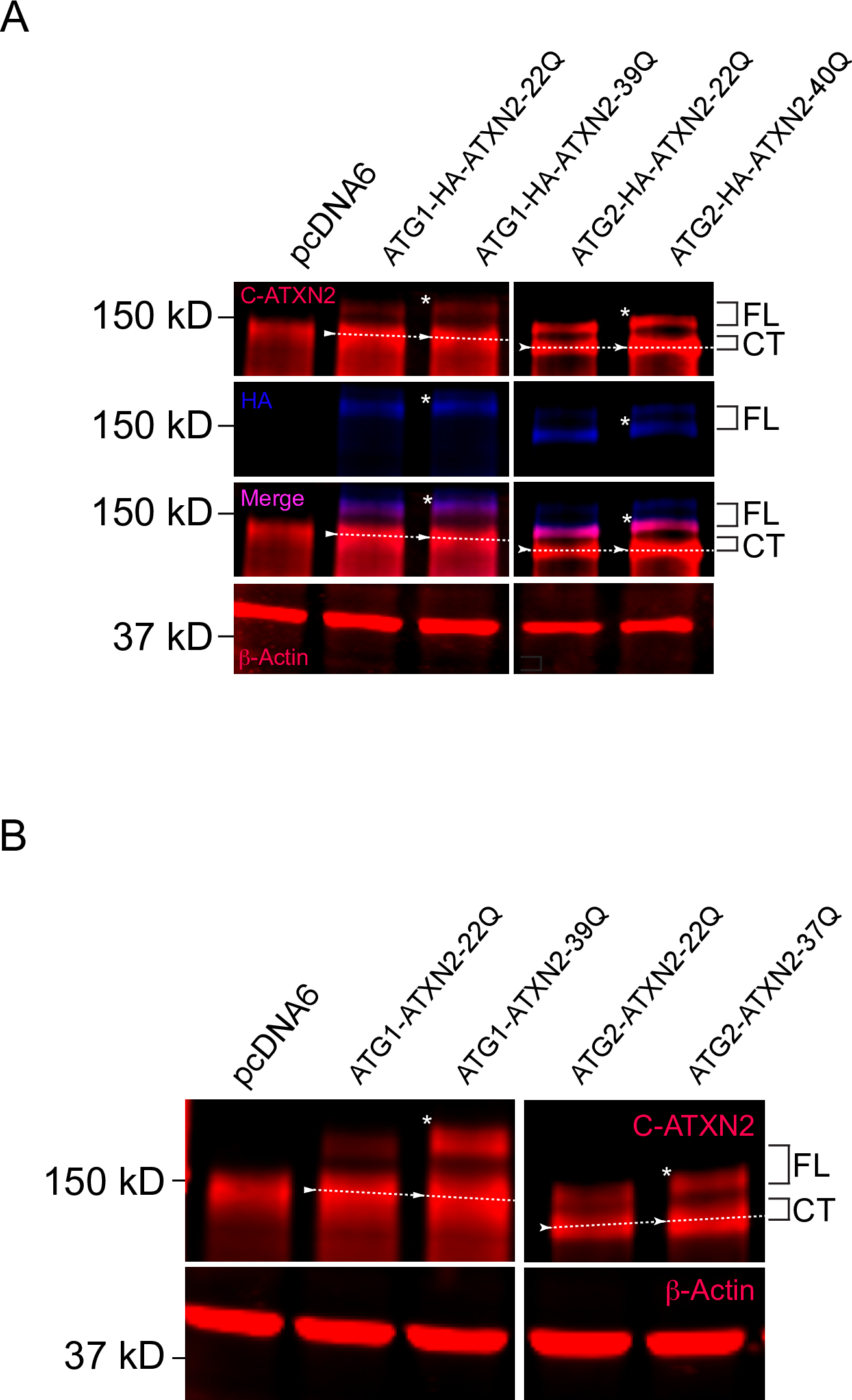

Supplement: S3 Fig — A. An additional representative blot illustrating the differing molecular weights of the HA-ATG1 vs. HA-ATG2 C-terminal fragments. HEK293E cells were transfected with plasmids shown in Fig 5A, and cell lysates were analyzed by immunoblotting. Red: Anti-C-ATXN2 or β-Actin, Blue: Anti-HA, Magenta: co-localized bands. FL = full-length ATG1-HA-ATXN2 and ATG2-HA-ATXN2 protein; CT = C-terminal fragment. * signs indicate the slower migration of mutant full-length ATXN2 proteins; Arrowheads and dotted lines, positioned in the middle of bands, emphasize migration difference between ATG1-HA-ATXN2 and ATG2-HA-ATXN2 C-terminal fragments. Flat arrowheads: ATG1-HA-ATXN2 C-terminal fragment; Curved arrowheads: ATG2-HA-ATXN2 C-terminal fragment. A Western blot representative of three independent experiments is shown. B. An additional representative blot illustrating the differing molecular weights of the ATG1 vs. ATG2 C-terminal fragments. HEK293E cells were transfected with plasmids shown in Fig 5C, and cell lysates were analyzed by immunoblotting. Red: Anti-C-ATXN2 or β-Actin, FL = full-length ATG1-ATXN2 and ATG2-ATXN2 protein; CT = C-terminal fragment. * signs indicate the slower migration of mutant full-length ATXN2 proteins; Arrowheads and dotted lines, positioned in the middle of bands, emphasize migration difference between ATG1-ATXN2 and ATG2-ATXN2 C-terminal fragments. Flat arrowheads: ATG1-ATXN2 C-terminal fragment; Curved arrowheads: ATG2-ATXN2 C-terminal fragment. A Western blot representative of three independent experiments is shown. (TIF) [file pone.0296085.s003.tif]
