# Supplementary material for: ATXN2 is a target of N-terminal proteolysis
Source: PLoS One. 2023 Dec 21;18(12):e0296085. doi: 10.1371/journal.pone.0296085 (PMC10735043; doi:10.1371/journal.pone.0296085)
Supplement: S1 Raw images — (PDF) [file pone.0296085.s004.pdf]

Figure 1

B

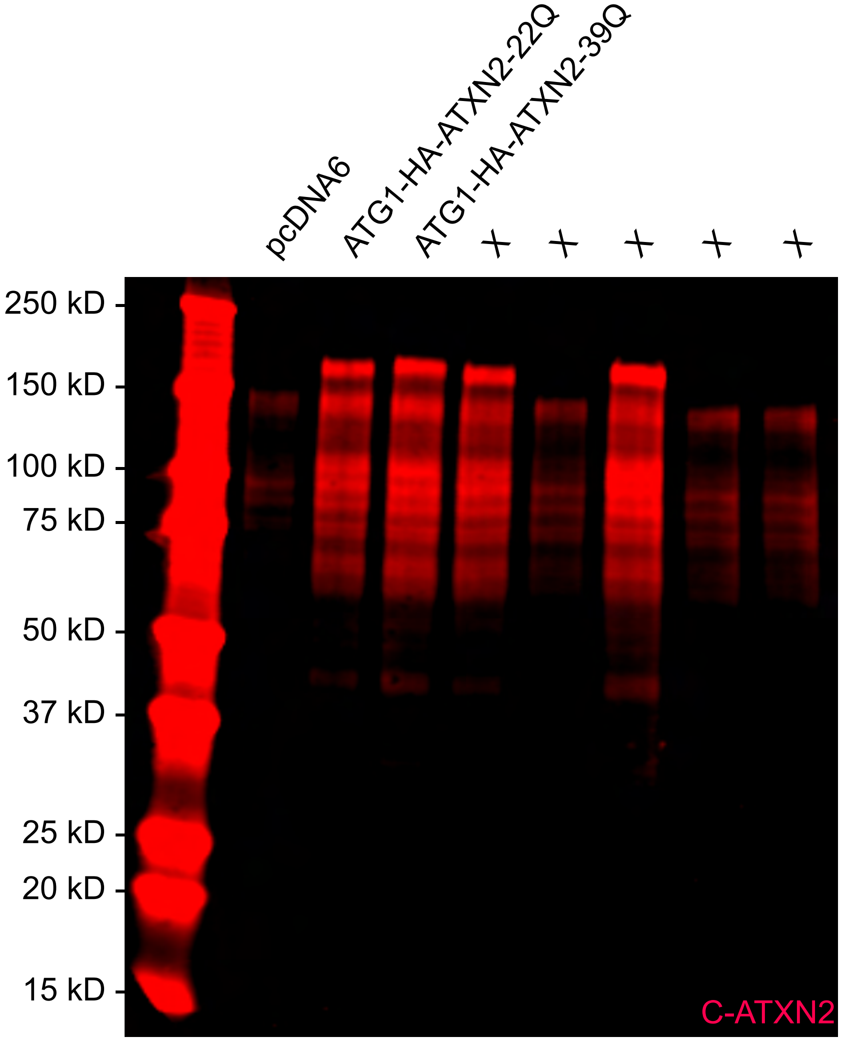
pcDNA6
ATG1-HA-ATXN2-22Q
ATG1-HA-ATXN2-39Q
X
X
X
X
X
250 kD
150 kD
100 kD
75 kD
50 kD
37 kD
25 kD
20 kD
15 kD
C-ATXN2

B

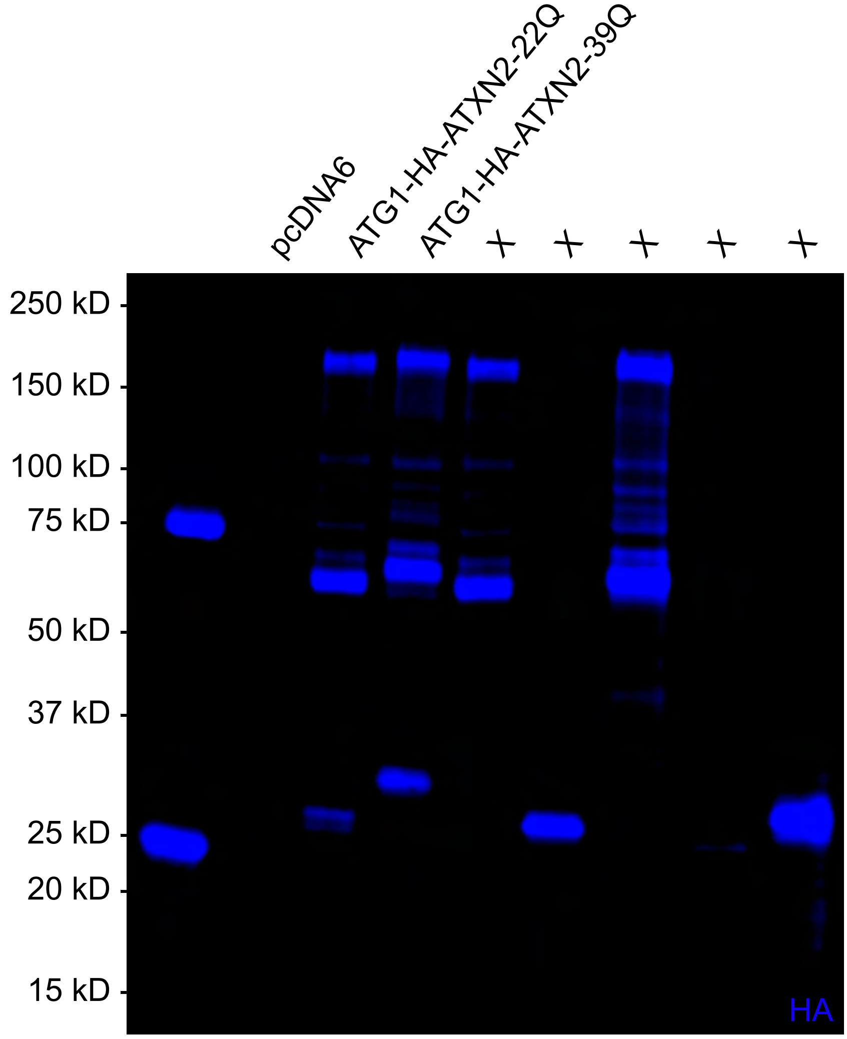
pcDNA6
ATG1-HA-ATXN2-22Q
ATG1-HA-ATXN2-39Q
X
X
X
X
X
250 kD
150 kD
100 kD
75 kD
50 kD
37 kD
25 kD
20 kD
15 kD
HA

B

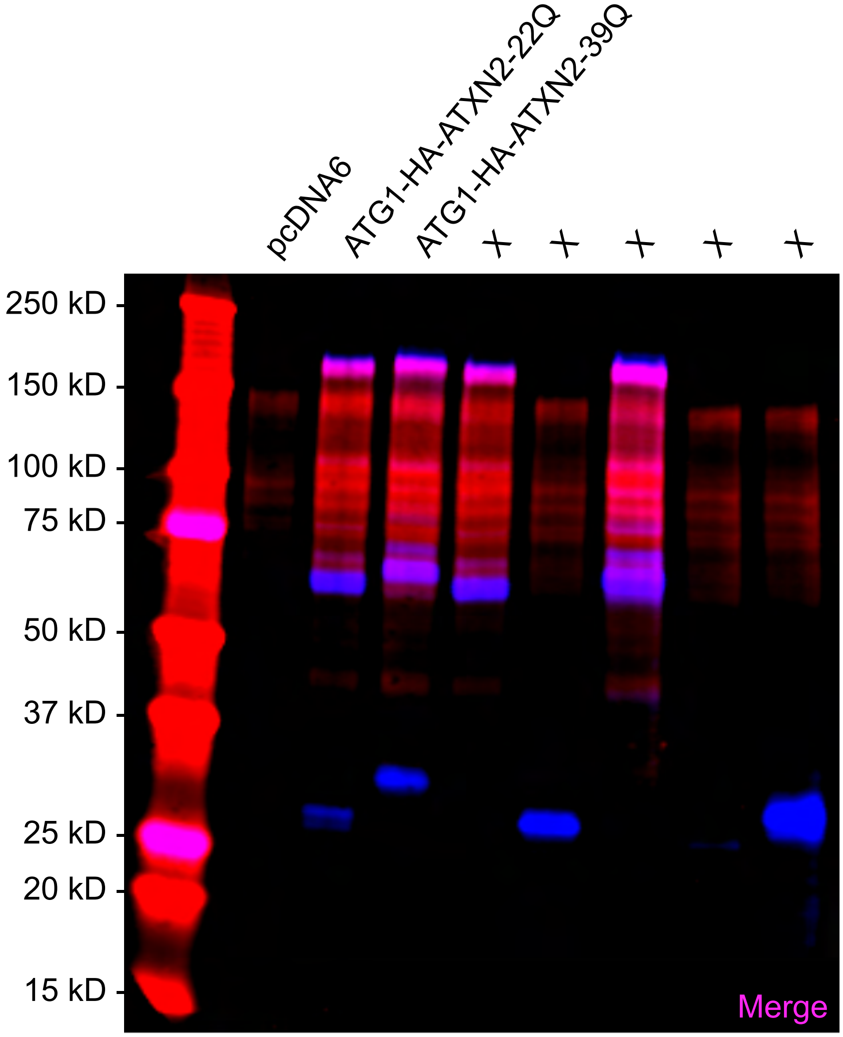
pcDNA6
ATG1-HA-ATXN2-22Q
ATG1-HA-ATXN2-39Q
X
X
X
X
X
250 kD
150 kD
100 kD
75 kD
50 kD
37 kD
25 kD
20 kD
15 kD
Merge

Figure 1

C

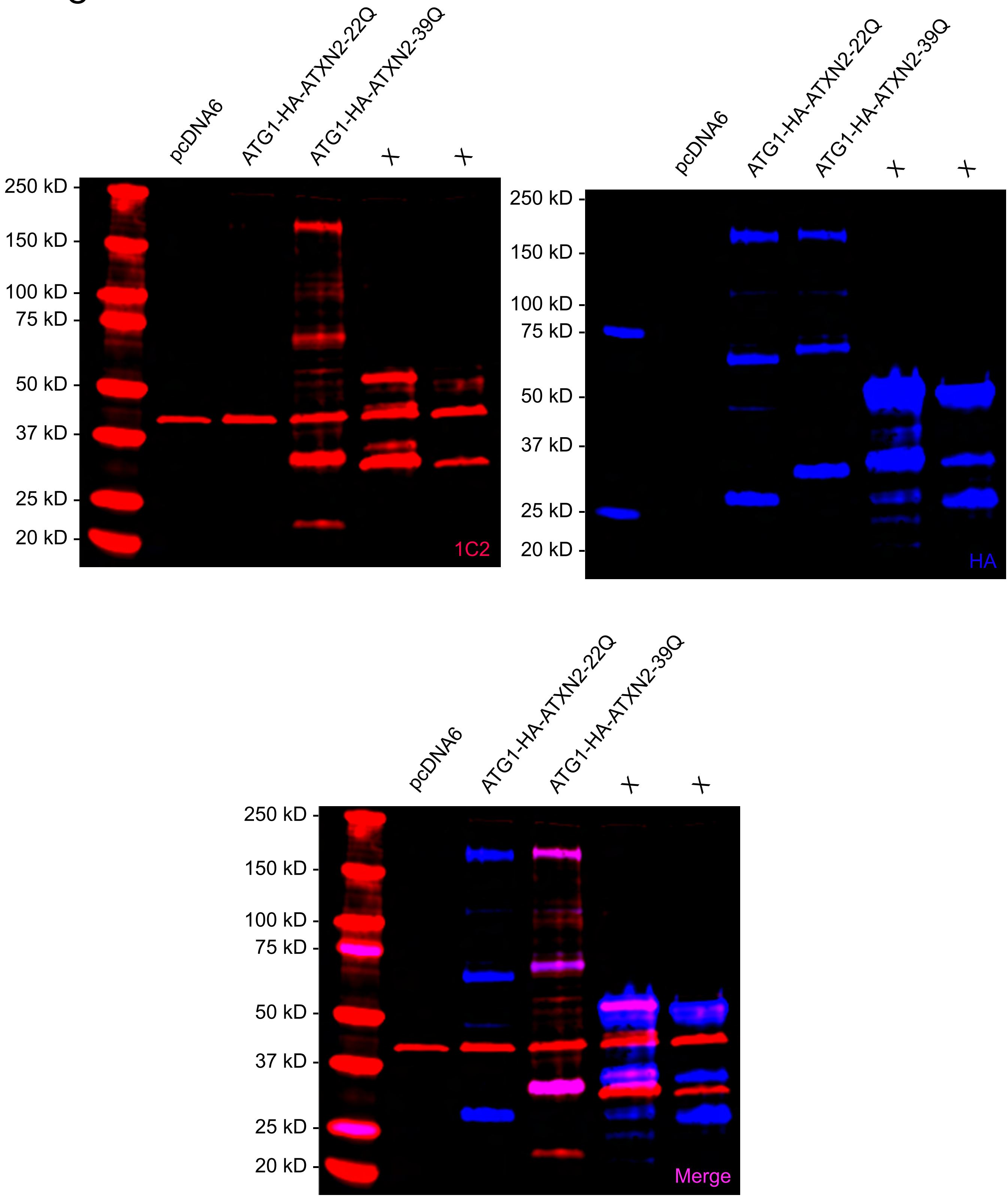
pcDNA6
ATG1-HA-ATXN2-22Q
ATG1-HA-ATXN2-39Q
X
X
250 kD
150 kD
100 kD
75 kD
50 kD
37 kD
25 kD
20 kD
1C2
HA
Merge

Figure 1

D

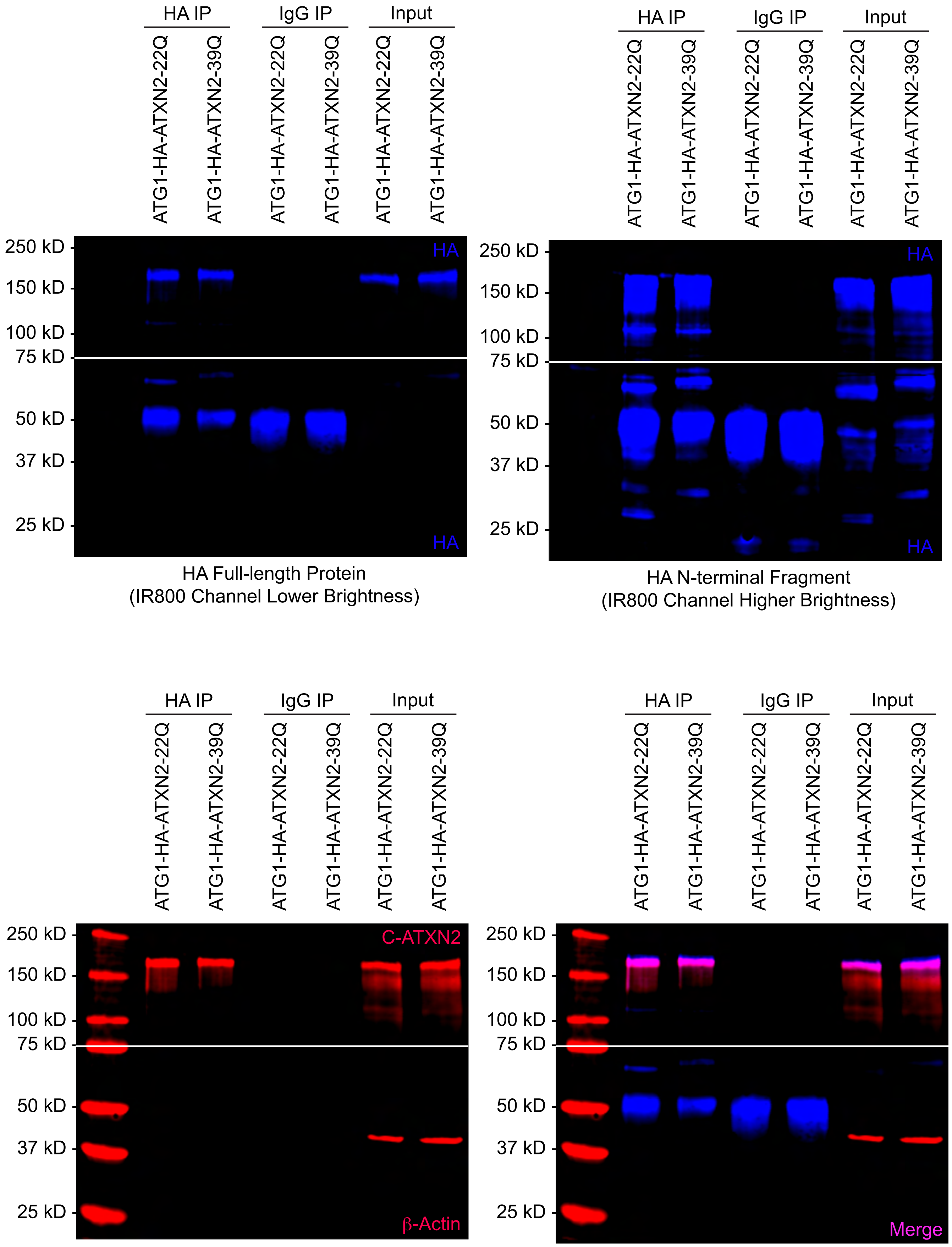
HA IP
IgG IP
Input
ATG1-HA-ATXN2-22Q
ATG1-HA-ATXN2-39Q
ATG1-HA-ATXN2-22Q
ATG1-HA-ATXN2-39Q
ATG1-HA-ATXN2-22Q
ATG1-HA-ATXN2-39Q
250 kD
150 kD
100 kD
75 kD
50 kD
37 kD
25 kD
HA
HA
HA Full-length Protein
(IR800 Channel Lower Brightness)
HA IP
IgG IP
Input
ATG1-HA-ATXN2-22Q
ATG1-HA-ATXN2-39Q
ATG1-HA-ATXN2-22Q
ATG1-HA-ATXN2-39Q
ATG1-HA-ATXN2-22Q
ATG1-HA-ATXN2-39Q
250 kD
150 kD
100 kD
75 kD
50 kD
37 kD
25 kD
HA
HA
HA N-terminal Fragment
(IR800 Channel Higher Brightness)
HA IP
IgG IP
Input
ATG1-HA-ATXN2-22Q
ATG1-HA-ATXN2-39Q
ATG1-HA-ATXN2-22Q
ATG1-HA-ATXN2-39Q
ATG1-HA-ATXN2-22Q
ATG1-HA-ATXN2-39Q
250 kD
150 kD
100 kD
75 kD
50 kD
37 kD
25 kD
C-ATXN2
β-Actin
HA IP
IgG IP
Input
ATG1-HA-ATXN2-22Q
ATG1-HA-ATXN2-39Q
ATG1-HA-ATXN2-22Q
ATG1-HA-ATXN2-39Q
ATG1-HA-ATXN2-22Q
ATG1-HA-ATXN2-39Q
250 kD
150 kD
100 kD
75 kD
50 kD
37 kD
25 kD
Merge

Figure 2

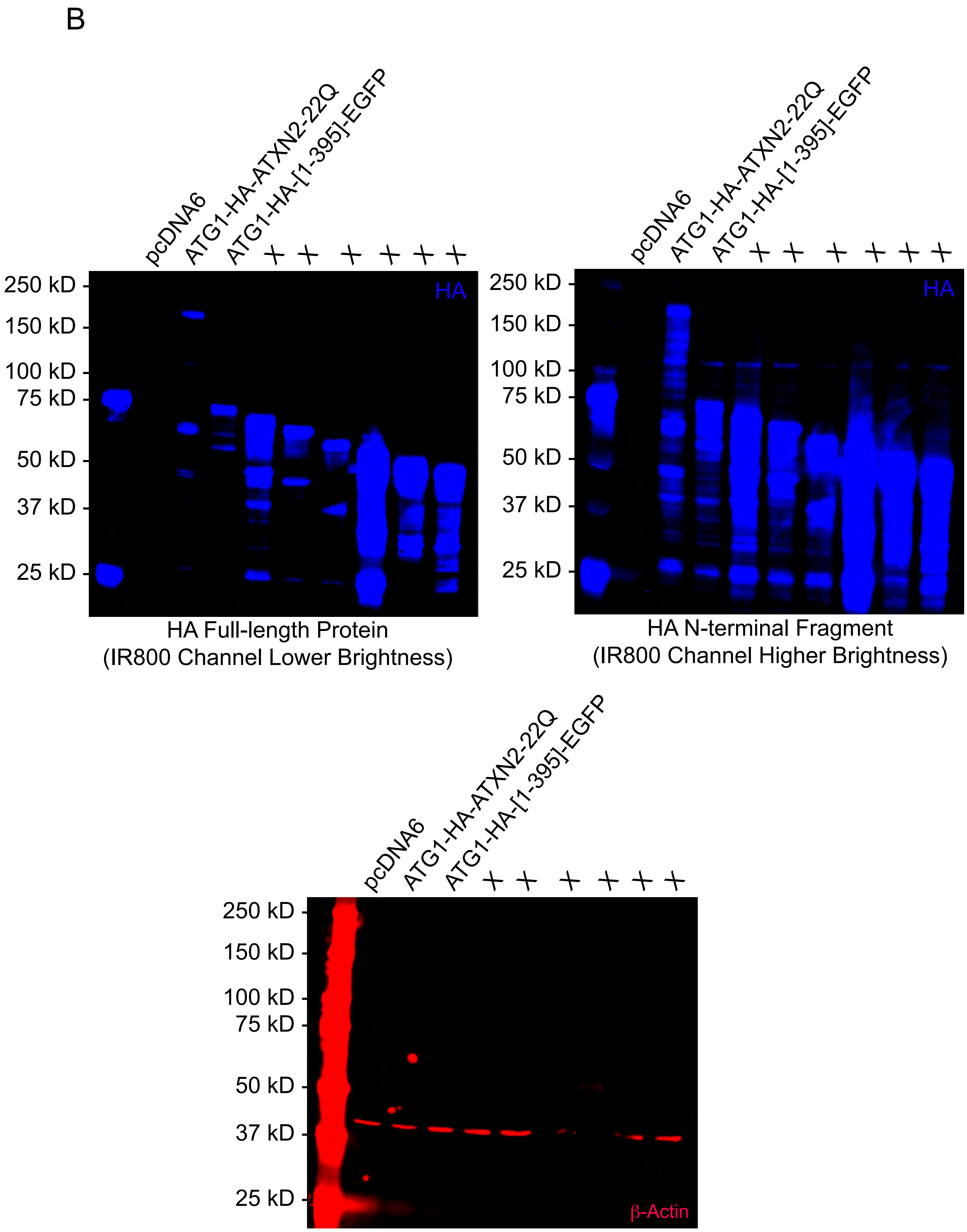
B
pcDNA6
ATG1-HA-ATXN2-22Q
ATG1-HA-[1-395]-EGFP
250 kD
150 kD
100 kD
75 kD
50 kD
37 kD
25 kD
HA
HA Full-length Protein
(IR800 Channel Lower Brightness)
HA N-terminal Fragment
(IR800 Channel Higher Brightness)
β-Actin

Figure 2

C

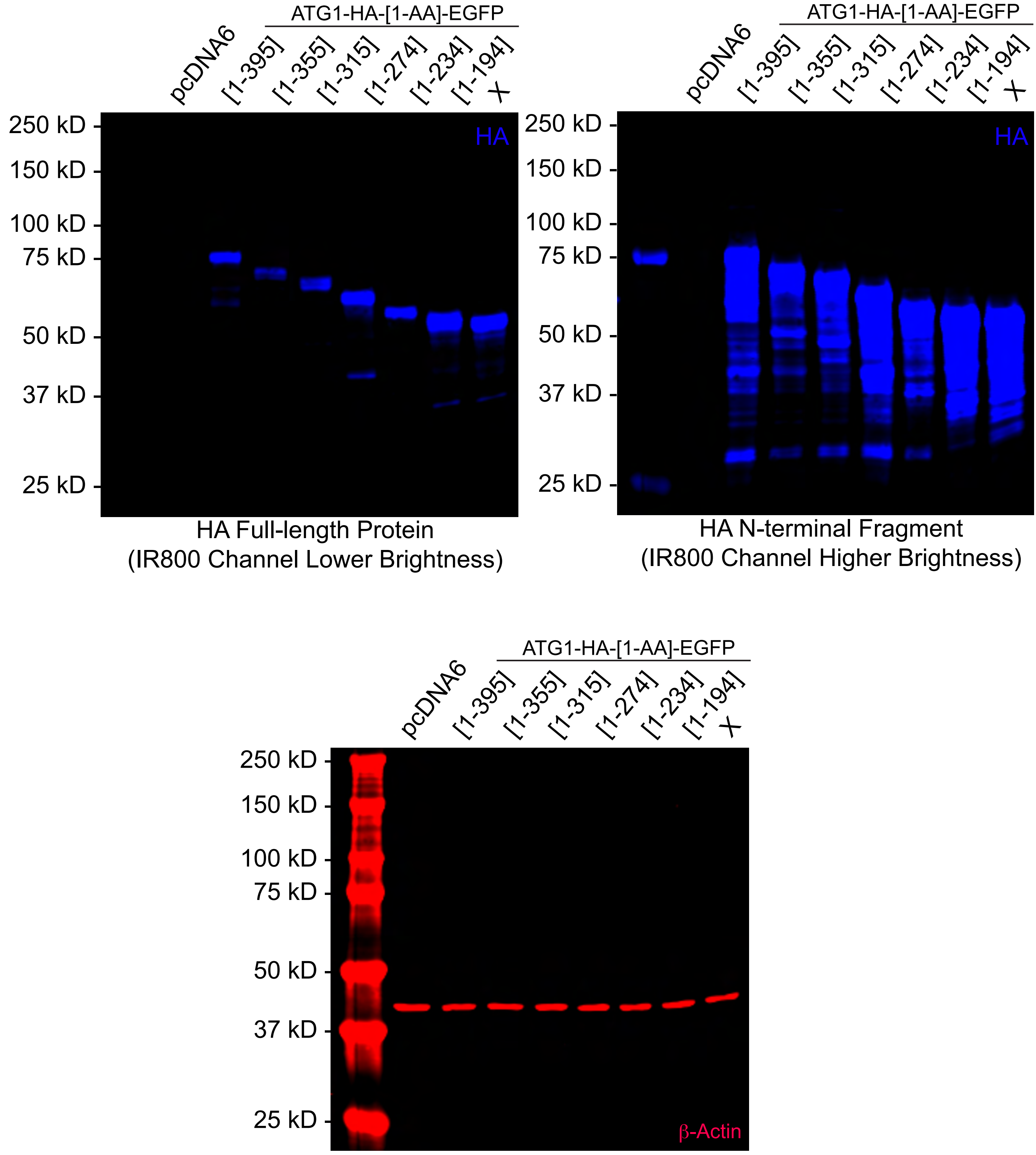

ATG1-HA-[1-AA]-EGFP
pcDNA6
[1-395]
[1-355]
[1-315]
[1-274]
[1-234]
[1-194]
X
250 kD
150 kD
100 kD
75 kD
50 kD
37 kD
25 kD
HA
HA Full-length Protein
(IR800 Channel Lower Brightness)
ATG1-HA-[1-AA]-EGFP
pcDNA6
[1-395]
[1-355]
[1-315]
[1-274]
[1-234]
[1-194]
X
250 kD
150 kD
100 kD
75 kD
50 kD
37 kD
25 kD
HA
HA N-terminal Fragment
(IR800 Channel Higher Brightness)
ATG1-HA-[1-AA]-EGFP
pcDNA6
[1-395]
[1-355]
[1-315]
[1-274]
[1-234]
[1-194]
X
250 kD
150 kD
100 kD
75 kD
50 kD
37 kD
25 kD
β-Actin

E

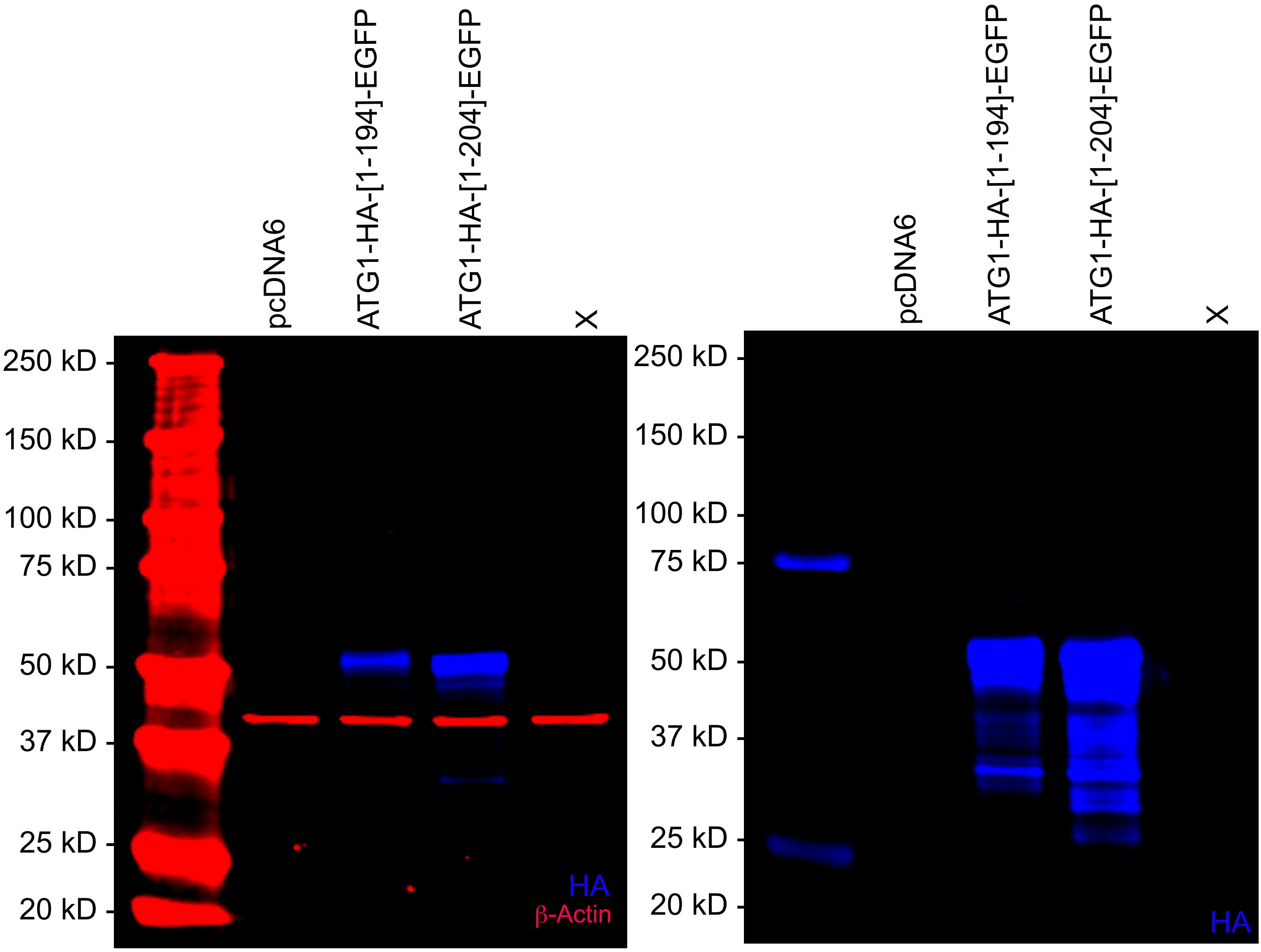

HA Full-length Protein
(IR800 Channel Lower Brightness)

HA N-terminal Fragment
(IR800 Channel Higher Brightness)

Figure 3

B

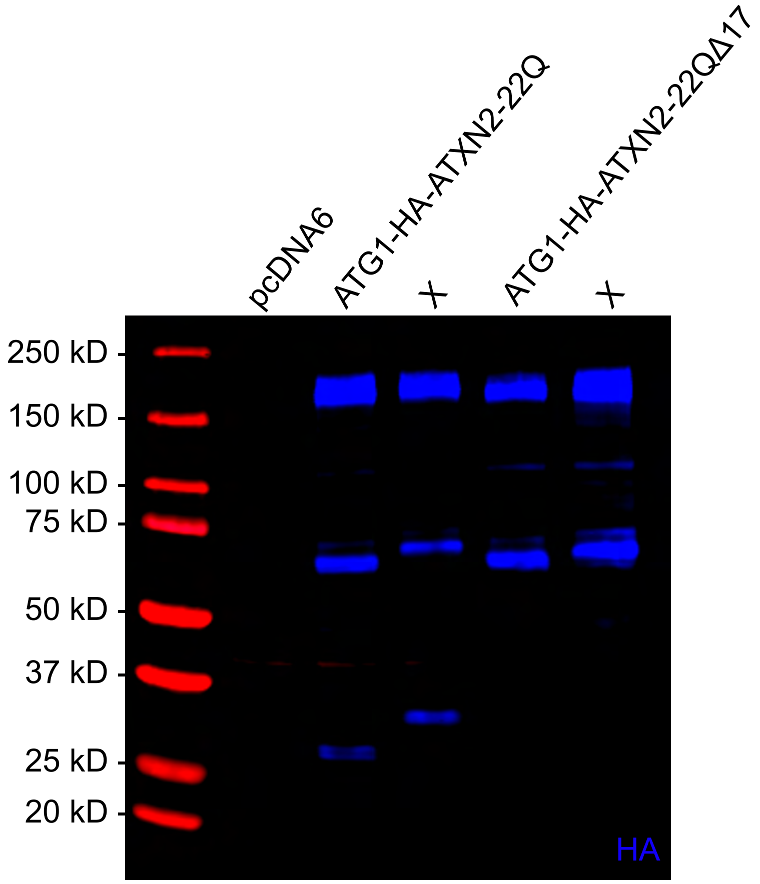
pcDNA6
ATG1-HA-ATXN2-22Q
X
ATG1-HA-ATXN2-22QΔ17
X
250 kD
150 kD
100 kD
75 kD
50 kD
37 kD
25 kD
20 kD
HA

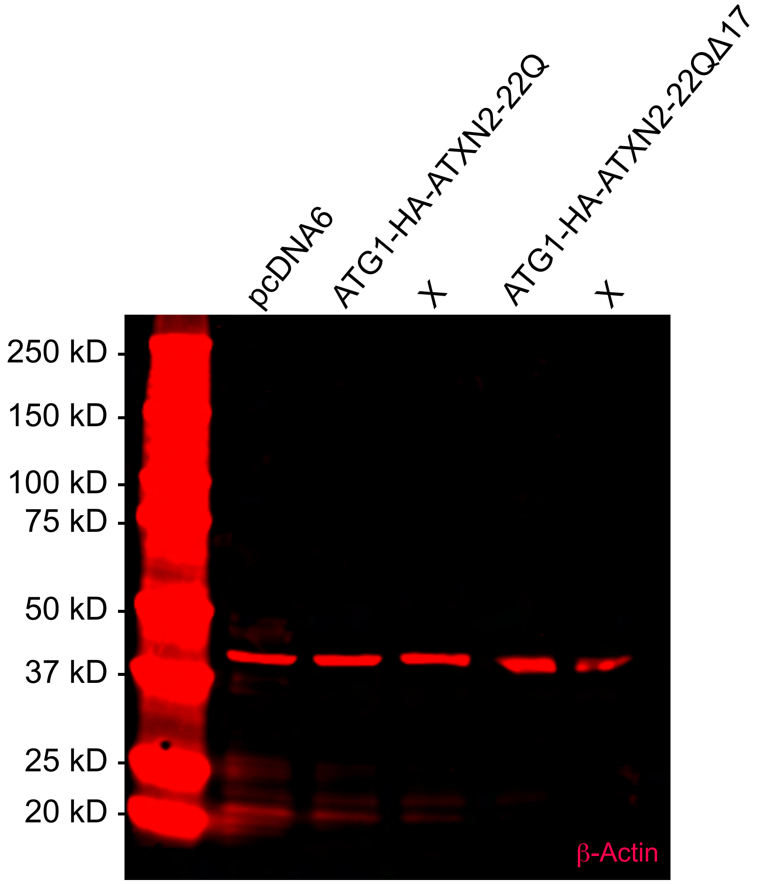
pcDNA6
ATG1-HA-ATXN2-22Q
X
ATG1-HA-ATXN2-22QΔ17
X
250 kD
150 kD
100 kD
75 kD
50 kD
37 kD
25 kD
20 kD
β-Actin

Figure 4

B

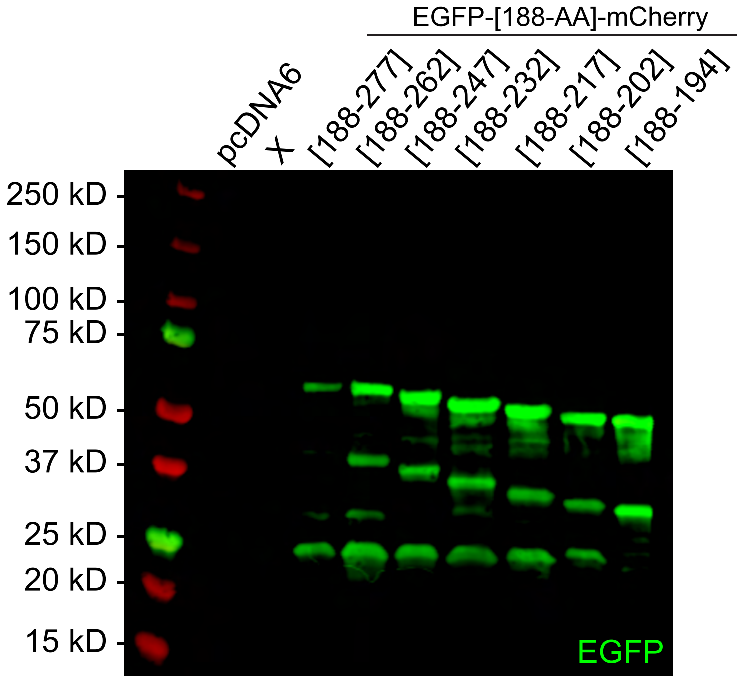
EGFP-[188-AA]-mCherry
pcDNA6
X
[188-277]
[188-262]
[188-247]
[188-232]
[188-217]
[188-202]
[188-194]
250 kD
150 kD
100 kD
75 kD
50 kD
37 kD
25 kD
20 kD
15 kD
EGFP

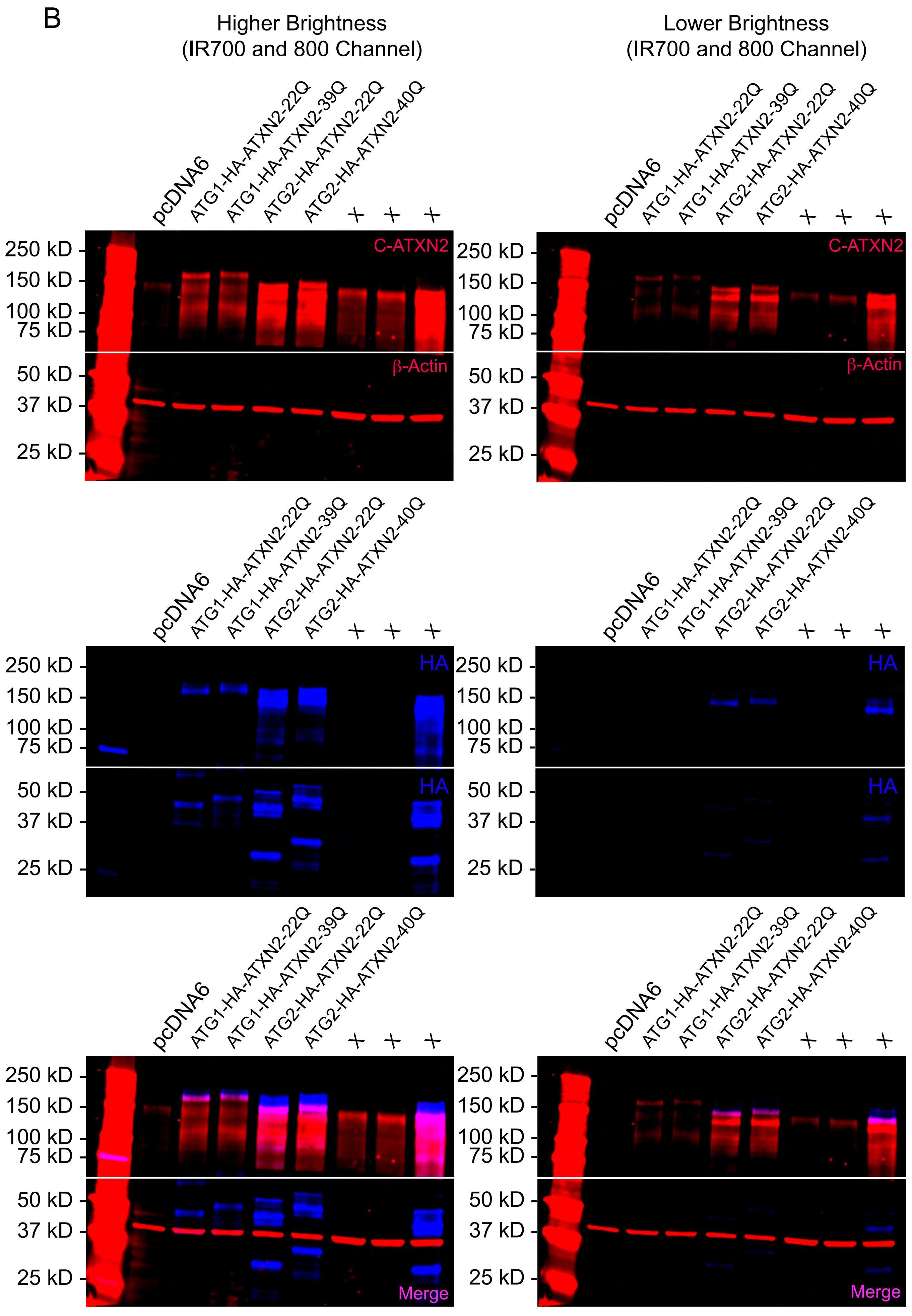
B
Higher Brightness
(IR700 and 800 Channel)
Lower Brightness
(IR700 and 800 Channel)
pcDNA6
ATG1-HA-ATXN2-22Q
ATG1-HA-ATXN2-39Q
ATG2-HA-ATXN2-22Q
ATG2-HA-ATXN2-40Q
X
X
X
250 kD
150 kD
100 kD
75 kD
50 kD
37 kD
25 kD
C-ATXN2
β-Actin
HA
Merge

Figure 5

D

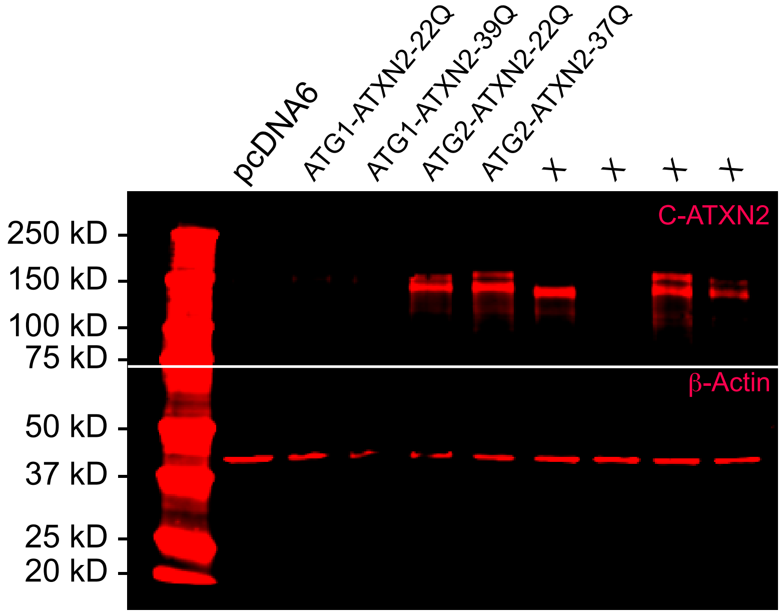

IR700 Channel Signal
(Lower Brightness)

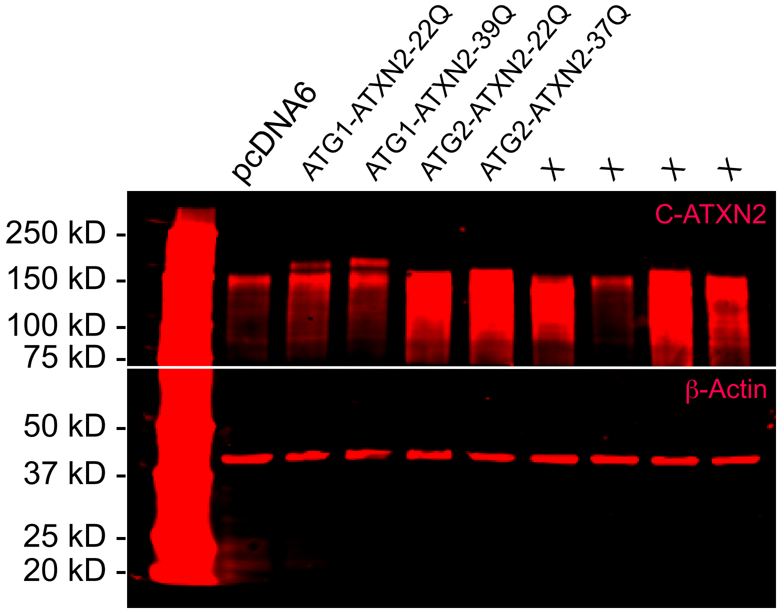

IR700 Channel Signal
(Higher Brightness)

Figure 6

B

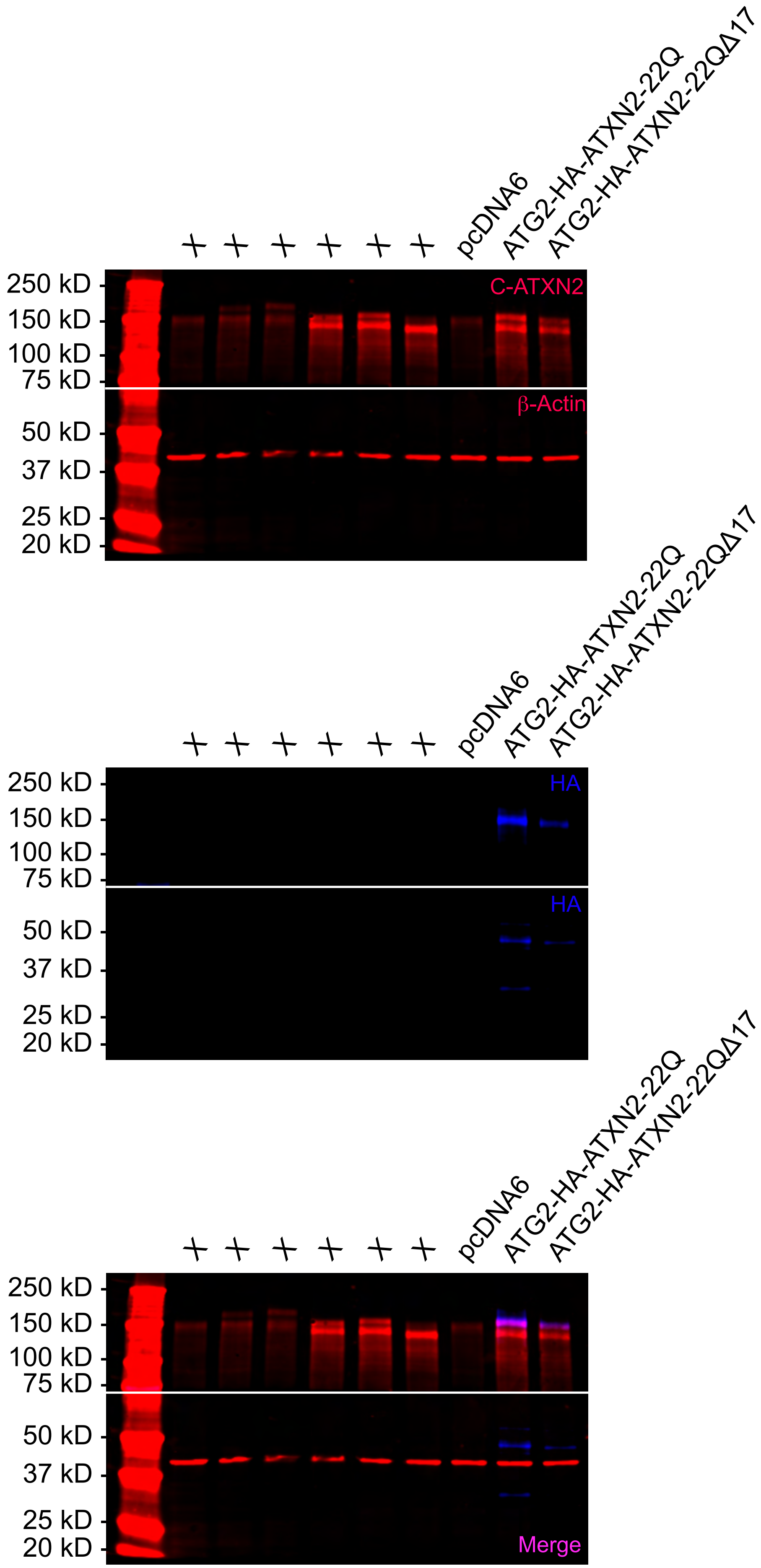

pcDNA6
ATG2-HA-ATXN2-22Q
ATG2-HA-ATXN2-22QΔ17
250 kD
150 kD
100 kD
75 kD
50 kD
37 kD
25 kD
20 kD
C-ATXN2
β-Actin
HA
HA
Merge

Figure S1

A

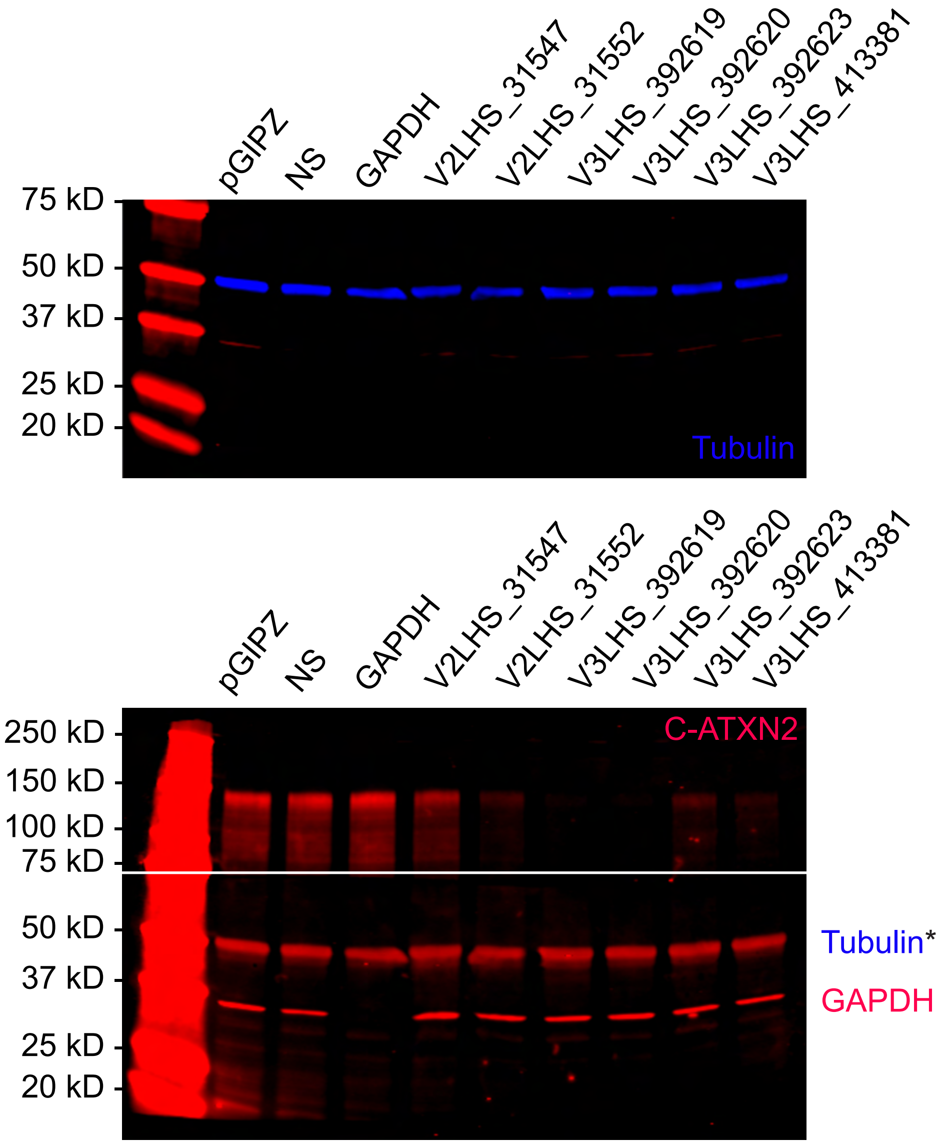

*Signal in IR700 channel due to cross-reactivity between rabbit anti-β-tubulin (Abcam #ab6046) and IRDye® 680RD goat anti-mouse IgG secondary antibody (Licor #926-68070)

Figure S2

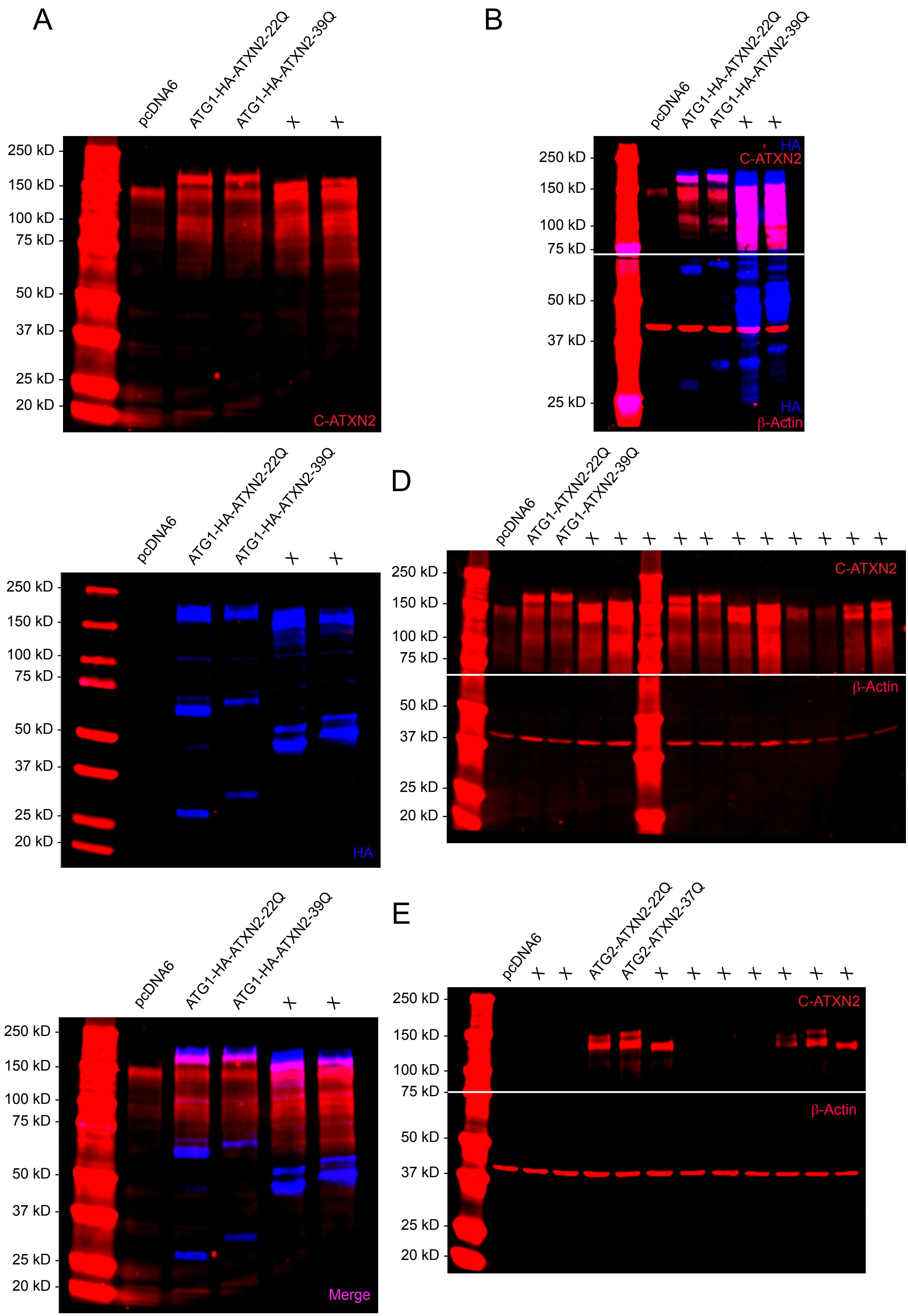
A
pcDNA6
ATG1-HA-ATXN2-22Q
ATG1-HA-ATXN2-39Q
250 kD
150 kD
100 kD
75 kD
50 kD
37 kD
25 kD
20 kD
C-ATXN2
B
HA
C-ATXN2
β-Actin
HA
D
ATG1-ATXN2-22Q
ATG1-ATXN2-39Q
E
ATG2-ATXN2-22Q
ATG2-ATXN2-37Q
Merge

Figure S3

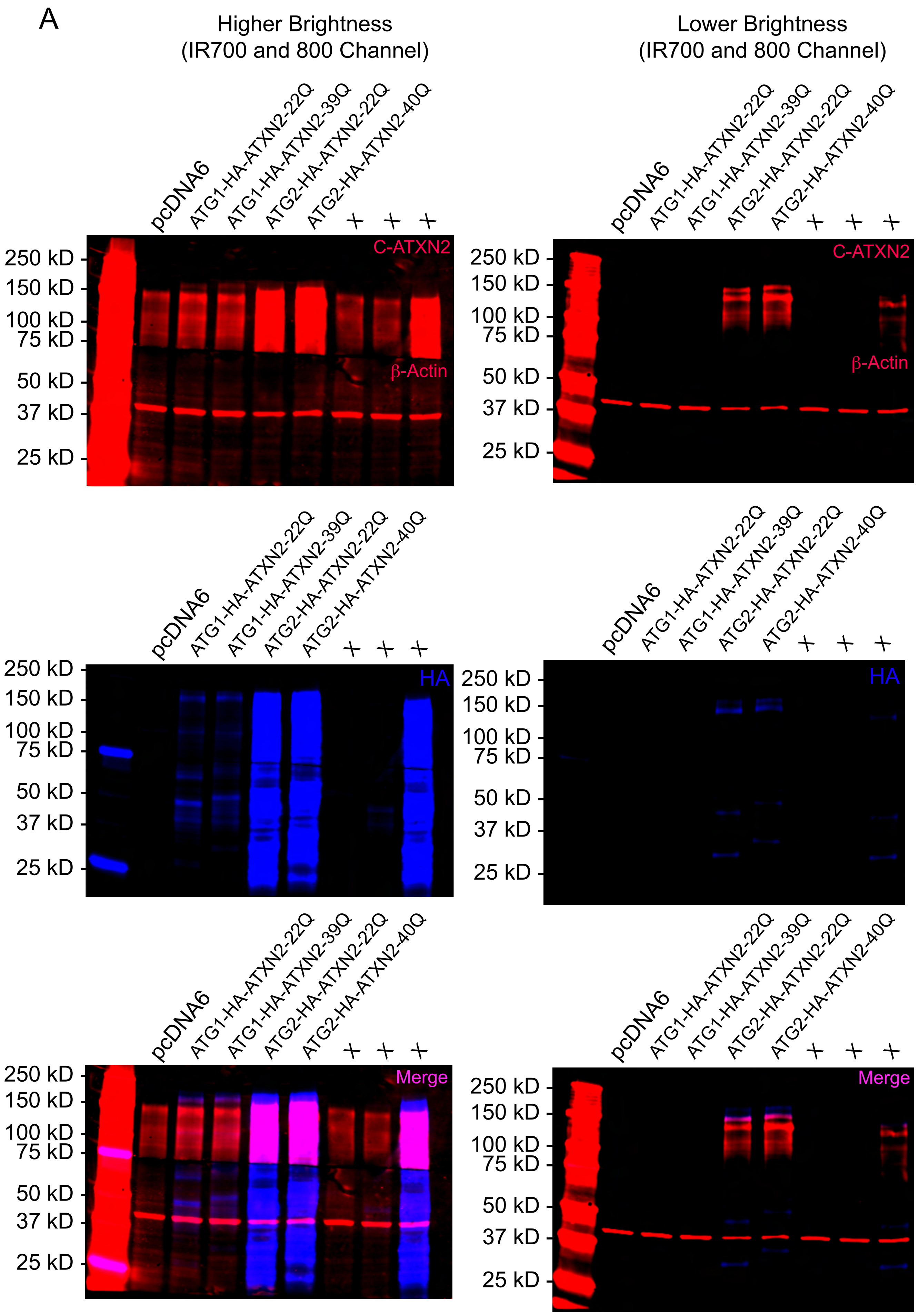
A
Higher Brightness
(IR700 and 800 Channel)
Lower Brightness
(IR700 and 800 Channel)
pcDNA6
ATG1-HA-ATXN2-22Q
ATG1-HA-ATXN2-39Q
ATG2-HA-ATXN2-22Q
ATG2-HA-ATXN2-40Q
X
X
X
250 kD
150 kD
100 kD
75 kD
50 kD
37 kD
25 kD
C-ATXN2
β-Actin
HA
Merge

B

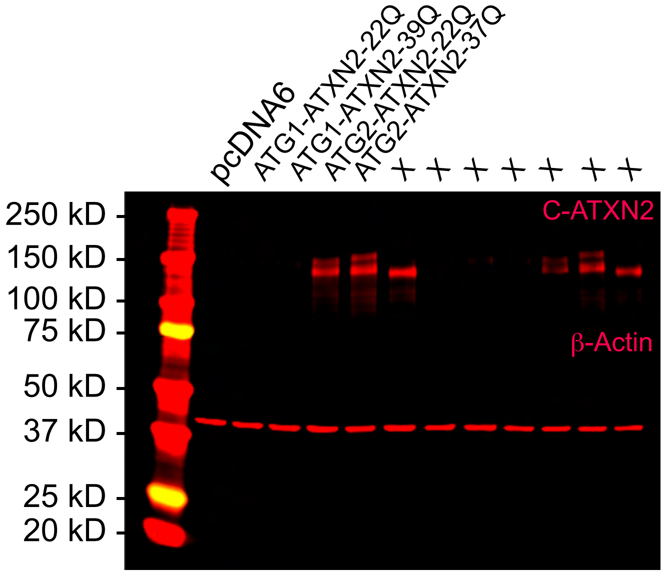

IR700 Channel Signal
(Lower Brightness)

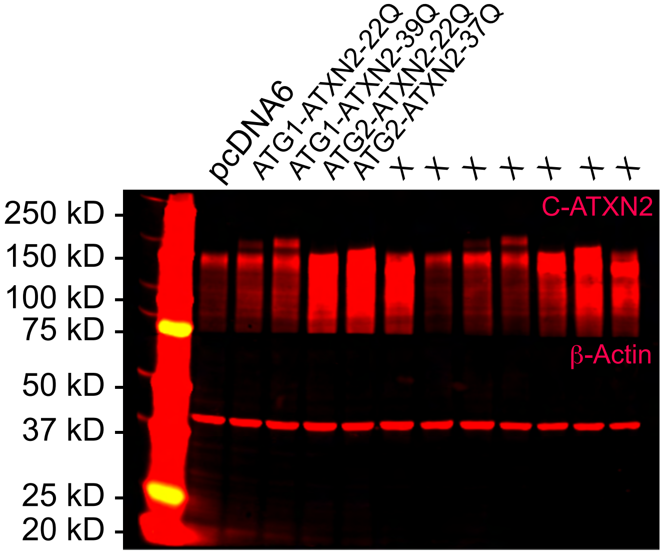

IR700 Channel Signal
(Higher Brightness)
